# A hybrid greedy political optimizer with fireworks algorithm for numerical and engineering optimization problems

**DOI:** 10.1038/s41598-022-17076-4

**Published:** 2022-08-02

**Authors:** Jian Dong, Heng Zou, Wenyu Li, Meng Wang

**Affiliations:** School of Computer Science and Engineering, Central South University, Changsha, China

**Keywords:** Mathematics and computing, Computer science, Engineering

## Abstract

This paper proposes a novel hybrid optimization algorithm named GPOFWA, which integrates political optimizer (PO) with fireworks algorithm (FWA) to solve numerical and engineering optimization problems. The original PO uses subgroup optimal solutions such as party leaders and constituency winners to guide the movement of the search agent. However, the number of such subgroup optimal solutions is limited, which leads to insufficient global exploration capabilities of PO. In addition, the recent past-based position updating strategy (RPPUS) of PO lacks effective verification of the updated candidate solutions, which reduces the convergence speed of the algorithm. The proposed hybrid algorithm uses the spark explosion mechanism in FWA to perform explosion spark and Gauss explosion spark operations on the subgroup optimal solutions (party leader and constituency winner) respectively based on the greedy strategy, which optimizes the subgroup optimal solution and enhances the exploitative ability of the algorithm. Moreover, Gaussian explosion sparks are also used to correct the candidate solutions after RPPUS, which makes up for the shortcomings of the original PO. In addition, a new subgroup optimal solution called the Converged Mobile Center (CMC) based on two-way consideration is designed to guide the movement of search agents and maintain the population diversity. We test the presented hybrid algorithm on 30 well-known benchmark functions, CEC2019 benchmark functions and three engineering optimization problems. The experimental results show that GPOFWA is superior to many statE−of-thE−art methods in terms of the quality of the resulting solution.

## Introduction

Optimization is a numerical process used to determine the decision variables for minimizing or maximizing the objective function value while satisfying the constraints of decision-space^1^. Optimization problems are inevitable in many real-world applications, and these problems usually contain non-linear objective functions and constraints with multiple local optimum, and low feasible regions^2^. These complex features make it difficult for traditional mathematical programming methods such as conjugate gradient, sequential quadratic programming, Newton's method, and quasi-Newton's method to find optimum^3^. Meta-heuristic algorithms (MAs) have become prevalent in many applied disciplines in recent decades because of higher performance and lower required computing capacity and time than deterministic algorithms in various optimization problems^4–12^. As a branch of random optimization, meta-heuristic algorithms can find a near-optimal solution by using available resources, although it is not always guaranteed to find the global optimum. Most MAs are inspired by human intelligence, the social nature of biological groups, and the laws of natural phenomena. Some classic representatives of MAs, such as genetic algorithm (GA)^13^, particle swarm optimization (PSO)^14^, differential evolution (DE)^15^, grey wolf optimizer (GWO)^16^, Harris hawks optimizer (HHO)^17^, bat algorithm (BA)^18^, whale optimization algorithm (WOA)^19^, salp swarm algorithm (SSA)^20^, sine cosine algorithm (SCA)^21^, water cycle algorithm (WCA)^22^, and so on, have been successfully used to solve some complex optimization problems.

However, the No Free Lunch (NFL) theorem states that it is impossible to solve all optimization problems by a specific algorithm^23^, which means an algorithm is suitable for a given optimization problem, but may not be suitable for another optimization problem with different characteristics. Therefore, further research on MAs is needed to deal with different optimization problems. The research directions of MAs include proposing new algorithms, improving existing algorithms, and hybridizing different algorithms. Hybridizing different algorithms has drawn attention because it can highlight their respective advantages and make the algorithms have better performance. Various hybrid algorithms have achieved good results, such as hybridizing particle swarm optimization with differential evolution proposed by Wang et al.^24^, hybridizing sine–cosine algorithm with differential evolution proposed by Li et al.^25^, hybridizing particle swarm with grey wolf optimizer presented by Zhang et al.^26^. Fireworks algorithm (FWA) was a newly developed swarm intelligence optimization algorithm, which was put forward by simulating the process of real fireworks explosion and generating a large number of sparks in 2010^27^. When the fireworks explode, the sparks are everywhere. The explosion process of the fireworks can be regarded as the search behavior of the search agent in the local space. The main idea of FWA is to use fireworks and sparks as different kinds of solutions to search the feasible space of the optimization function. As an excellent algorithm, FWA has been used in hybridization with many other algorithms in recent years. Zhu et al.^28^ hybridized the firework algorithm with the particle swarm algorithm to form DFWPSO, which performed competitively and effectively in numerical optimization problems. Yue et al.^29^ proposed a new hybrid algorithm called FWGWO based on gray wolf optimizer and firework algorithm and achieved excellent results in global optimization. Guo et al.^30^ added the differential evolution operator to the firework algorithm and proposed a hybrid fireworks algorithm with differential evolution operator (HFWA_DE) in 2019. Zhang et al.^31^ introduced the migration operator of biogeography-based optimization into fireworks algorithm to enhance information sharing among populations and presented a hybrid biogeography-based optimization and fireworks algorithm for global optimization.

Political Optimizer (PO) is a new meta-heuristic algorithm based on human behavior inspired by the multi-stage political process. PO simulates all important steps in politics, such as party formation, party vote, constituency distribution, election campaigns, and party transitions, inter-party elections, and parliamentary affairs after the government is formed. In addition, PO has introduced a new position update strategy, called the recent past-based position updating strategy (RPPUS). The latter represents the behavior that politicians learned from the last election^32^. Compared with traditional optimization algorithms, PO shows better competitiveness. Therefore, lots of researchers have applied it in different scientific fields since the PO was proposed. Askari et al.^33^ employed PO for the training of feedforward neural networks to solve the classification and regression problems, and made a good achievement. Durmus et al. used PO to improve radiation properties of concentric circular antenna arrays (CCAAs) in the far-field such as wireless communication of smart grids and the Internet of things and reached a lower sidelobe level (SLL) value than other optimization methods^34^. Manita et al.^35^ proposed a binary version of PO to solve feature selection problems using gene expression data. Elsheikh et al.^36^ presented a novel optimized predictive model based on PO for eco-friendly MQL-turning of AISI 4340 alloy with nano-lubricants. Moreover, some scholars have made improvements to the shortcomings of PO. Askari et al.^37^ modified each stage of PO to improve the exploration ability and balance of the algorithm because it is found PO prematurely converges for complex problems. Zhu et al.^38^ also found that PO has the problem of poor global exploration capabilities, and they integrated PO with quadratic interpolation, advanced quadratic interpolation, cubic interpolation, Lagrange interpolation, Newton interpolation, and refraction learning, and proposed a sequence of novel PO variants.

As a novel swarm intelligence algorithm just proposed, PO still has many areas worth improving. It can be found that the main idea of PO is to guide the movement of the search agent through subgroup optimal solutions. However, the number of subgroup optimal solutions such as party leaders and constituency winners used by PO is limited, because the number of initial populations directly determines the number of party leaders and constituency winners. This leads to insufficient global exploration capabilities of PO. In addition, the recent past-based position updating strategy (RPPUS) of PO lacks effective verification of the updated candidate solutions, which reduces the convergence speed of the algorithm. Moreover, a new local leader called the Converged Mobile Center (CMC) based on two-way consideration is designed to guide the movement of search agents, which enhances the exploration ability and maintains the population diversity. Combining the above ideas, we propose a novel hybrid greedy political optimizer with fireworks algorithm named GPOFWA and verify its effectiveness and superiority through a well-studied set of diverse benchmark functions and three engineering optimization problems. In summary, the main contributions of this research are as follows:

1. We propose a new hybrid optimization algorithm named GPOFWA, which integrates the Political Optimizer (PO) and the Fireworks Algorithm (FWA). Using the spark explosion mechanism in FWA, GPOFWA performs explosion spark and Gaussian explosion spark operations on party leaders and constituency winners based on greedy strategy, which enhances exploitation capability of GPOFWA. At the same time, the Gaussian explosion spark mechanism of the firework algorithm is used to explore areas with better fitness to ensure the effectiveness of RPPUS.
2. We adopt a new method called Converged Mobility Center with bi-directional consideration to generate the subgroup optimal solution of the current population, which enhances the exploration ability and maintains the population diversity.
3. We investigate the performance of the proposed algorithm in solving 30 basic benchmark functions in multiple dimensions (30 and 500), CEC2019 benchmark functions and three engineering optimization problems. To verify the feasibility and effectiveness of this scheme and the accuracy of the results from different aspects, we use experimental and statistical analysis, such as qualitative analysis, quantitative analysis, convergence preference, pairwise comparative analysis (Wilcoxon signed-rank test), computational complexity, and sensitivity analysis of parameters.

The remainder of this research is organized as follows: Section 2 reviews the basic political optimizer and fireworks algorithm. Section 3 proposes a novel hybrid greedy political optimizer with fireworks algorithm. Section 4 discusses the experiment results of different swarm intelligence optimization algorithms on basic benchmark functions and CEC2019 functions. Section 5 applies the algorithm to three different engineering optimization problems. Section 6 presents the conclusions of this work and directions for future work.

## Related work

Political optimizer and firework algorithm are novel algorithms with excellent performance proposed in recent years, which are inspired by different social natural phenomena and can effectively solve optimization problems. The hybrid algorithm proposed in this paper takes the political optimization algorithm as the starting point, and the explosion spark and Gaussian mutation spark mechanism of the firework algorithm are added to the search process of the political optimization algorithm to enhance the performance of the algorithm. This section will briefly introduce these two algorithms.

### Political optimizer

**Figure 1:**
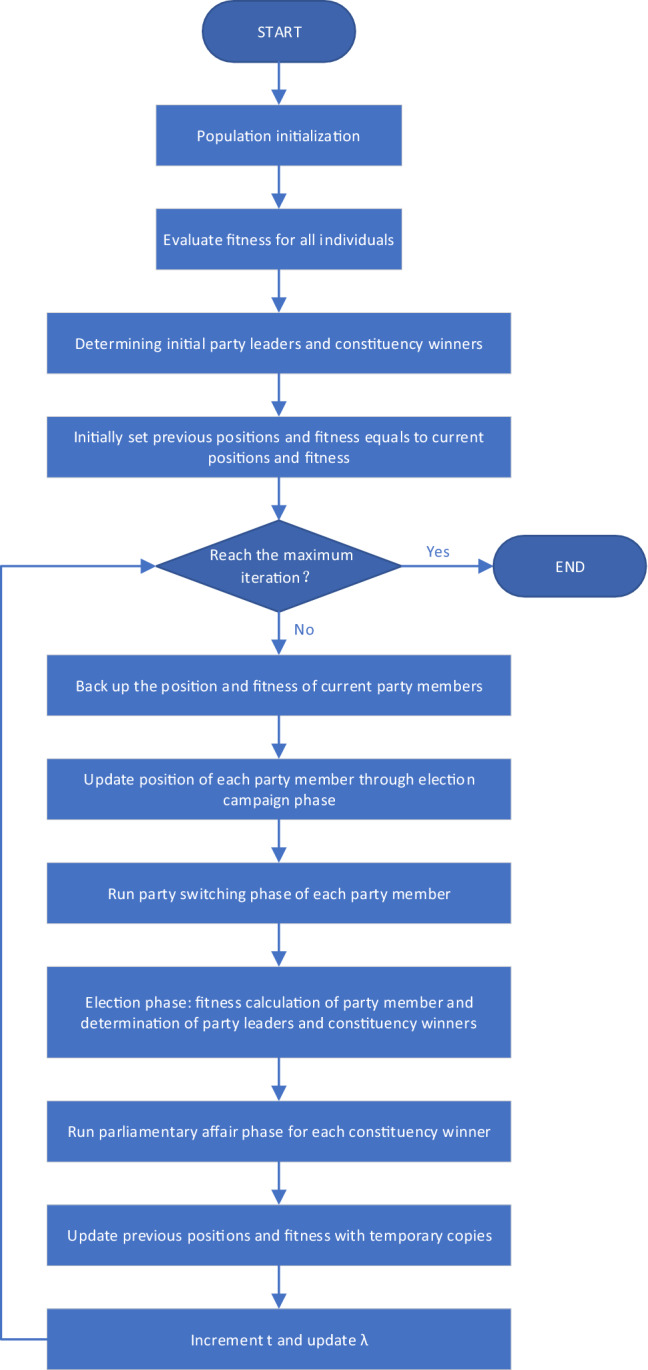
The flowchart of political optimizer.

The political optimizer (PO) is a novel intelligent optimization algorithm inspired by the political election process of human society. In PO, each party member can be viewed as a candidate solution, and the election behavior of party members can be seen as an evaluation function. In addition, the votes obtained by party members are mapped to the fitness value of the candidate solution. Unlike traditional algorithms based on political elections, PO considers the complete process of political elections, including five phases of party formation and constituency allocation, election campaign, party switching, inter-party election, and parliamentary affairs. PO seeks the optimal solution through a multi-stage iterative process, and its main algorithm flow is shown in Fig. 1. The following will introduce the five main stages of PO.

#### Party formation and constituency allocation

**Figure 2:**
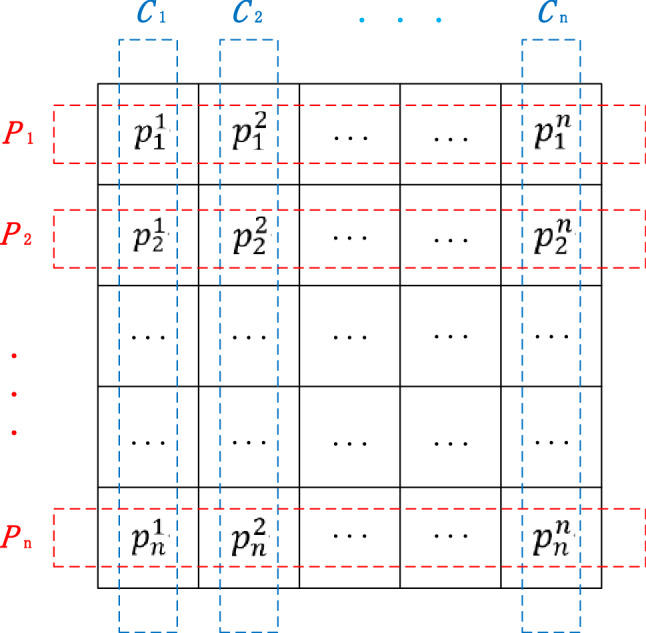
The population and its logical division in political parties and constituencies.

At the beginning of PO, the entire population containing ${n}^{2}$ individuals are divided into *n* parties, and there are *n* members (candidate solution) in each party. In addition, each party member also plays the role of an election candidate, that is, one member from each party is selected to form a constituency. As is depicted in Fig. 2, the red dotted line indicates the division of political parties, and the blue dotted line indicates the division of constituencies. The mapping of this population division to the mathematical model is that the entire population is divided into *n* political parties as shown in Eq. (1), and each party consists of *n* party members as represented as Eq. (2).

1 $$P = \left\{ {P_{1} ,P_{2} ,P_{3} , \ldots ,P_{n} } \right\}$$

2 $$P_{i} = \left\{ {p_{i}^{1} ,p_{i}^{2} ,p_{i}^{3} , \ldots ,p_{i}^{n} } \right\}$$

Each party member also plays the role of an election candidate, so the entire population can be regarded as *n* constituencies, which can be represented as Eq. (3). What needs to be emphasized is the members of the constituency are also party members, but the logical division is different. The membership of each constituency is divided as shown in Eq. (4).

3 $$C = \left\{ {C_{1} ,C_{2} ,C_{3} , \ldots ,C_{n} } \right\}$$

4 $$C_{j} = \left\{ {p_{1}^{j} ,p_{2}^{j} ,p_{3}^{j} , \ldots ,p_{n}^{j} } \right\}$$

Furthermore, the leader of the *i*th party after computing the fitness of all members is noted as $p_{i}^{*}$ and the set of all the party leaders is represented by ${P}^{*}$ as shown in Eq. (5). Similarly, after the election, ${C}^{*}$ regroups the winners from all the constituencies named the parliamentarians as shown in Eq. (6), where $c_{j}^{*}$ denotes the winner of *j*th constituency.

5 $$P^{*} = \left\{ {p_{1}^{*} ,p_{2}^{*} ,p_{3}^{*} , \ldots ,p_{n}^{*} } \right\}$$

6 $$C^{*} = \left\{ {c_{1}^{*} ,c_{2}^{*} ,c_{3}^{*} , \ldots ,c_{n}^{*} } \right\}$$

#### Election campaign

This stage is the core stage of the algorithm and is responsible for the location update of the search agent. In the algorithm, the specific performance is that party members change their positions according to the leader ${P}^{*}$ of the party they belong to and the winner ${C}^{*}$ of their constituency. In addition, they will also learn from the experience of the last election through a novel location update mechanism called recent past-based position updating strategy (RPPUS), as formulated in Eqs. (7) and (8). The main idea of RPPUS is to predict promising areas through the numerical relationship between subgroup optimal solution (party leader or constituency winner) and current fitness and previous fitness of search agent.

7 $$p_{i,k}^{j} \left( {t + 1} \right) = \left\{ {\begin{array}{ll} {m^{*} + r\left( {m^{*} - p_{i,k}^{j} (t)} \right),} \hfill & {{\text{if}}\quad p_{i,k}^{j} (t - 1) \le p_{i,k}^{j} (t) \le m^{*} {\text{ or }}p_{i,k}^{j} (t - 1) \ge p_{i,k}^{j} \left( t \right) \ge m^{*} } \hfill \\ {m^{*} + (2r - 1)\left| {m^{*} - p_{i,k}^{j} (t)} \right|,} \hfill & {{\text{if}}\quad p_{i,k}^{j} (t - 1) \le m^{*} \le p_{i,k}^{j} (t){\text{ or }}p_{i,k}^{j} (t - 1) \ge m^{*} \ge p_{i,k}^{j} (t)} \hfill \\ {m^{*} + (2r - 1)\left| {m^{*} - p_{i,k}^{j} (t - 1)} \right|,} \hfill & {{\text{if}}\quad m^{*} \le p_{i,k}^{j} (t - 1) \le p_{i,k}^{j} (t){\text{ or }}m^{*} \ge p_{i,k}^{j} (t - 1) \ge p_{i,k}^{j} (t)} \hfill \\ \end{array} } \right.$$

8 $$p_{i,k}^{j} \left( {t + 1} \right) = \left\{ {\begin{array}{ll} {m^{*} + \left( {2r - 1} \right)\left| {m^{*} - p_{i,k}^{j} \left( t \right)} \right|,} \hfill & {{\text{if}}\quad p_{i,k}^{j} \left( {t - 1} \right) \le p_{i,k}^{j} \left( t \right) \le m^{*} {\text{ or }}p_{i,k}^{j} \left( {t - 1} \right) \ge p_{i,k}^{j} \left( t \right) \ge m^{*} } \hfill \\ {p_{i,k}^{j} \left( {t - 1} \right) + r\left( {p_{i,k}^{j} \left( t \right) - p_{i,k}^{j} \left( {t - 1} \right)} \right),} \hfill & {{\text{if}}\quad p_{i,k}^{j} \left( {t - 1} \right) \le m^{*} \le p_{i,k}^{j} \left( t \right){\text{ or }}p_{i,k}^{j} \left( {t - 1} \right) \ge m^{*} \ge p_{i,k}^{j} \left( t \right)} \hfill \\ {m^{*} + \left( {2r - 1} \right)\left| {m^{*} - p_{i,k}^{j} \left( {t - 1} \right)} \right|,} \hfill & {{\text{if}}\quad m^{*} \le p_{i,k}^{j} \left( {t - 1} \right) \le p_{i,k}^{j} \left( t \right){\text{ or }}m^{*} \ge p_{i,k}^{j} \left( {t - 1} \right) \ge p_{i,k}^{j} \left( t \right)} \hfill \\ \end{array} } \right.$$

where $m^{*}$ indicates the leader of a party or the winner of a constituency, $r$ represents a random number from 0 to 1, and $t$ represents the current iteration number.

#### Party switching

The party switching phase is mainly to balance exploration and exploitation, which introduces an adaptive parameter $\lambda$ called party switching rate. Each party member may be selected and switched to some randomly selected party. The probability of switching is determined by $\lambda$, which is initially 1 and linearly decreases to 0 as shown in Eq. (9).

9 $$\lambda = \left( {1 - \frac{t}{T}} \right)*\lambda_{{{\text{max}}}}$$

#### Election

At this stage, the fitness of each candidate solution is determined and the party leaders and constituency winners are updated by Eqs. (10) and (11).

10 $$q = \mathop {{\text{argmin}}}\limits_{1 \leqslant j \leqslant n} f\left( {p_{i}^{j} } \right)\quad p_{i}^{*} = p_{i}^{q}$$

11 $$q = \mathop {{\text{argmin}}}\limits_{1 \leqslant i \leqslant n} f\left( {p_{i}^{j} } \right)\quad c_{j}^{*} = p_{q}^{j}$$

#### Parliamentary affairs

The party switching phase is aimed at the change of the party's perspective, and the parliamentary affairs phase is the change of the constituency's perspective. The constituency winners interact with each other to improve their fitness. Each constituency winner uses the following equation to update its position relative to any other randomly selected constituency. It should be noted that the movement will only be applied if the fitness of $c_{j}^{*}$ becomes better.

### Fireworks algorithm

**Figure 3:**
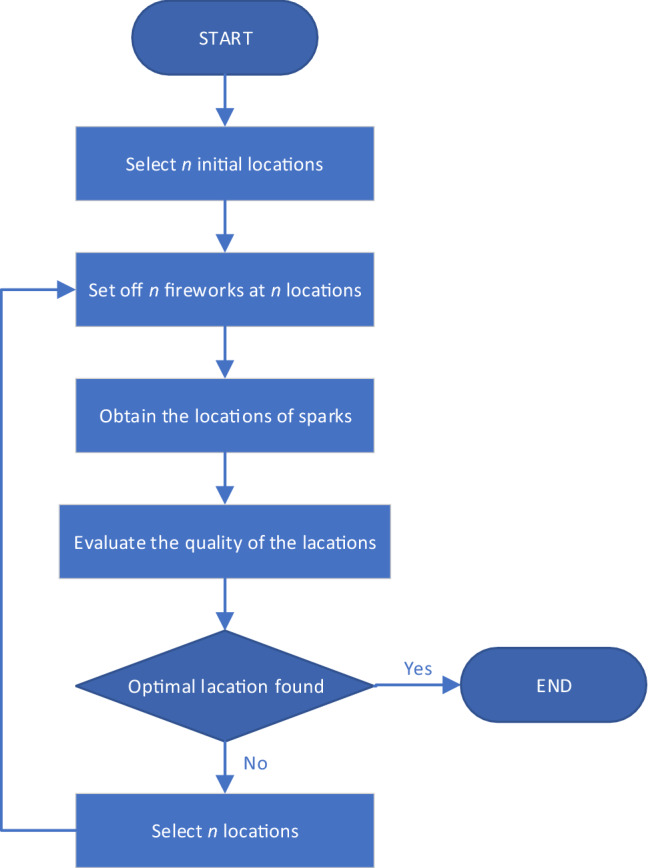
The flowchart of firework algorithm.

The firework algorithm (FWA) is a swarm intelligence optimization algorithm proposed in recent years, which is inspired by the explosion of fireworks. We usually celebrate with fireworks. When the fireworks explode, the sparks are everywhere. The explosion process of the fireworks can be regarded as the search behavior of the search agent in the local space. The firework algorithm is based on this idea, and the flowchart of the firework algorithm is shown in Fig. 3.

It should be emphasized that fireworks of different qualities will produce different sparks when they explode. High-quality fireworks will produce countless sparks when they explode. The explosion of the fireworks forms a circle, and the sparks are concentrated in the center of the explosion. Conversely, a bad firework will produce fewer sparks when it explodes, and the sparks will spread out to form irregular shapes. From the perspective of swarm intelligence algorithm, a firework is regarded as a candidate solution. A good firework means that a candidate solution is located in a promising area and is close to the global optimal solution. Therefore, more sparks can be generated near good fireworks to find the global optimal solution, and the search radius is as small as possible. A bad firework means that the position of the candidate solution is not ideal, so the search radius should be larger, and the number of sparks generated will be reduced accordingly.

As mentioned earlier, good fireworks should produce more sparks, while bad fireworks produce fewer sparks. The calculation of the number of sparks produced by each firework is shown in Eq. (12). Good fireworks are closer to the global optimum, so the explosion amplitude is smaller, while bad fireworks are just the opposite. The amplitude of explosion for each firework is defined as Eq. (13).

12 $$S_{i} = \hat{S} \cdot \frac{{y_{{{\text{max}}}} - f({\varvec{x}}_{{\varvec{i}}} ) + \xi }}{{\mathop \sum \nolimits_{i = 1}^{n} (y_{{{\text{max}}}} - f({\varvec{x}}_{{\varvec{i}}} )) + \xi }}$$

13 $$A_{i} = \hat{A} \cdot \frac{{f({\varvec{x}}_{{\varvec{i}}} ) - y_{{{\text{min}}}} + \xi }}{{\mathop \sum \nolimits_{i = 1}^{n} (f({\varvec{x}}_{{\varvec{i}}} ) - y_{{{\text{min}}}} ) + \xi }}$$

where $y_{{{\text{min}}}} = {\text{min}}(f({\varvec{x}}_{{\varvec{i}}} ))$, $y_{{{\text{max}}}} = {\text{max}}(f({\varvec{x}}_{{\varvec{i}}} ))$, $\hat{S}$ and $\hat{A}$ are constants, which are to control the number of explosion sparks and the size of explosion amplitude, respectively.

What should be noted is FA design two ways of generating sparks, one is explosion sparks for normal search, its algorithm is shown in Algorithm 1. The other is Gaussian spark, which is a mutation mechanism, and its algorithm is shown in Algorithm 2.
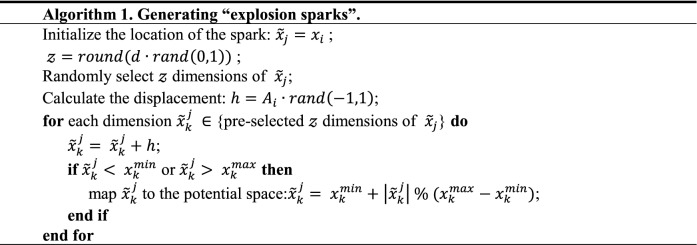



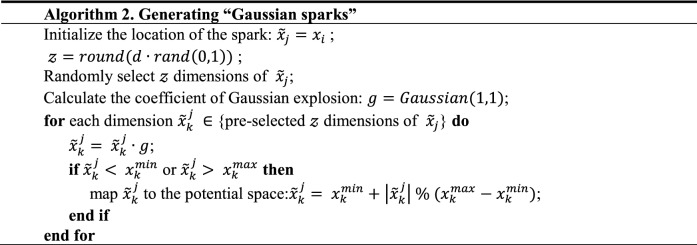


## Proposed method

The original PO assigns dual roles to each agent and uses RPPUS to make the algorithm have excellent performance, but through careful observation, we can find that the algorithm still has a lot of room for improvement. There are the following several points:

1. The main idea of PO is to guide the movement of the search agent through the subgroup optimal solution. The number of subgroup optimal solutions such as party leader and constituency winner are limited because the number of initial populations directly determines the number of party leaders and constituency winners, which leads to insufficient global exploration capabilities of PO.
2. In RPPUS, member positions are updated based on the positions of members of the previous generation, the positions of party leaders or constituency winners, and the current positions of members. Considering the numerical relationship between these three indicators, it effectively predicts the favorable area of the member's next move, but this is the future movement trend predicted based on only three indicators, and its accuracy needs to be improved. Moreover, after the update is completed, it is not verified whether the fitness has improved.
3. In the position update process, to consider the influence of the party leader and the constituency winner on the position of the members, the members are successively moved around the two subgroup optimal solutions. If the two subgroup optimal solutions themselves are relatively close, the difference between updating twice and updating once is not large, and updating twice also means that all dimensions of each member must be updated twice, which adds a lot of time consumption.

The proposed algorithm puts forward corresponding solutions based on the above points, and finally forms GPOFWA. For the first point, using the spark explosion mechanism in FWA, GPOFWA performs explosion spark and Gauss explosion spark operations on party leader and constituency winner respectively based on greedy strategy, thereby optimizing the subgroup optimal solution. For the second point, GPOFWA uses the Gaussian explosion spark mechanism of the firework algorithm to explore areas with better adaptability to ensure the effectiveness of RPPUS. Regarding the third point, this article proposes a new subgroup optimal solution, called Converged Mobility Center (CMC) with bi-directional consideration, which not only considers the advantages of the party leader and the constituency winner but also maintains the population diversity.

### Hybridizing political optimizer with fireworks algorithm

**Figure 4:**
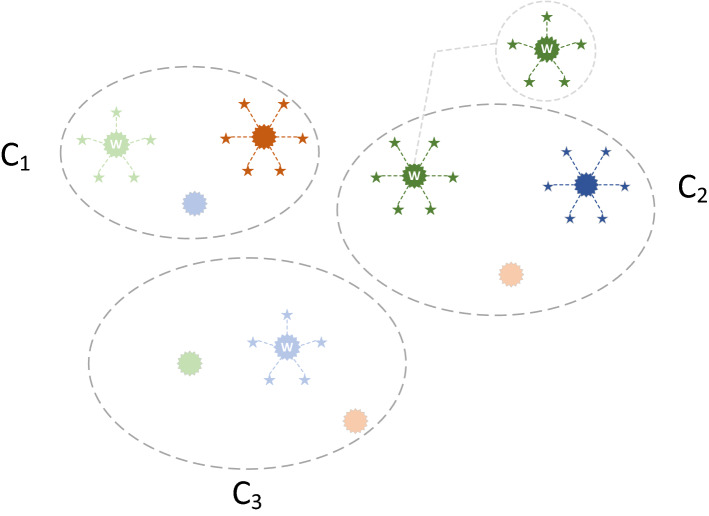
Party leaders and constituency winners perform explosion operation.

The most distinctive feature of FWA is that the firework explosion operator truly simulates the search process of the search agent. Generating a large number of sparks means that a large number of candidate solutions are generated. PO updates the position of search agent around subgroup optimal solutions, but the number of subgroup optimal solutions is limited by the size of the initial population. At the same time, the individuals performing the explosion operation in FWA are selected optimally from the entire population, and subgroup optimal solution of PO has been screened out, which can be used for the explosion operation. Moreover, the two explosion methods of FWA correspond to the two subgroup optimal solutions of PO, and they complement each other. Here, the party leaders conduct the explosion spark operation, and the constituency winners conduct the Gaussian spark operation. The detailed process of their explosive operation is shown in Fig. 4. In the figure, each dot represents a candidate solution, and each fivE−pointed star represents the spark produced by the explosion. Dots of the same color indicate that they belong to the same political party, and the darkest colored dots indicate the leader of the party. Obviously, the dots in the same ellipse belong to a constituency, and the dots marked with a “W” letter indicate the winner of the constituency. The leader of the party conducts an explosion spark operation (hexagonal firework), while the constituency winner conducts a Gaussian explosion operation (pentagonal firework).

Similar to the FWA, the calculation of the number of sparks generated by subgroup optimal solution is shown in Eqs. (14) and (15). The difference is that in the process of generating sparks, only the subgroup optimal solutions are considered. A better subgroup optimal solution generates more sparks, and a lower fitness subgroup optimal solution generates fewer sparks.

14 $$K_{i}^{p} = k \cdot \frac{{p_{{{\text{max}}}}^{*} - f\left( {p_{i}^{*} } \right) + \xi }}{{\mathop \sum \nolimits_{i = 1}^{N} \left( {p_{{{\text{max}}}}^{*} - f\left( {p_{i}^{*} } \right)} \right) + \xi }}$$

15 $$K_{j}^{c} = k \cdot \frac{{c_{{{\text{max}}}}^{*} - f\left( {c_{j}^{*} } \right) + \xi }}{{\mathop \sum \nolimits_{j = 1}^{N} \left( {c_{{{\text{max}}}}^{*} - f\left( {c_{j}^{*} } \right)} \right) + \xi }}$$

where $K_{i}^{p}$ indicates the number of sparks generated by the leader of the $ith$ party, $K_{j}^{c}$ indicates the number of sparks generated by the winner of the $jth$ constituency, *k* is a parameter controlling the total number of sparks generated by party leaders or constituency winners, $p_{{{\text{max}}}}^{*} = {\text{max}}\left( {f\left( {p_{i}^{*} } \right)} \right)$ ($i = 1, 2, \ldots , N$) is the maximum (worst) value of the objective function among the *N* party leaders, $c_{{{\text{max}}}}^{*} = {\text{max}}\left( {f\left( {c_{j}^{*} } \right)} \right)$ ($j = 1, 2, \ldots , N$) is the maximum (worst) value of the objective function among the *N* constituency winners, and $\xi$, which denotes the smallest constant in the computer, is utilized to avoid zero-division-error.

Since the party leaders conduct the explosion spark operation, it is necessary to calculate the explosion range. The calculation formula is shown as Eq. (16).

16 $$R_{i}^{p} = R \cdot \frac{{f\left( {p_{i}^{*} } \right) - p_{{{\text{min}}}}^{*} + \xi }}{{\mathop \sum \nolimits_{i = 1}^{N} \left( {f\left( {p_{i}^{*} } \right) - p_{{{\text{min}}}}^{*} } \right) + \xi }}$$

where $R_{i}^{p}$ represents the explosion range of the leader of the $ith$ party, *R* denotes the maximum explosion range, $p_{{{\text{min}}}}^{*} = {\text{min}}\left( {f\left( {p_{i}^{*} } \right)} \right)$ ($i = 1, 2, \ldots , N$) is the minimum (best) value of the objective function among the *N* party leaders.

It should be noted that after the party leaders and the constituency winners perform the explosion operation, based on the greedy strategy, they will update themselves if the sparks they generate have better fitness than themselves. This process is carried out after party formation and constituency allocation, whose pseudo-code is shown in Algorithm 3.
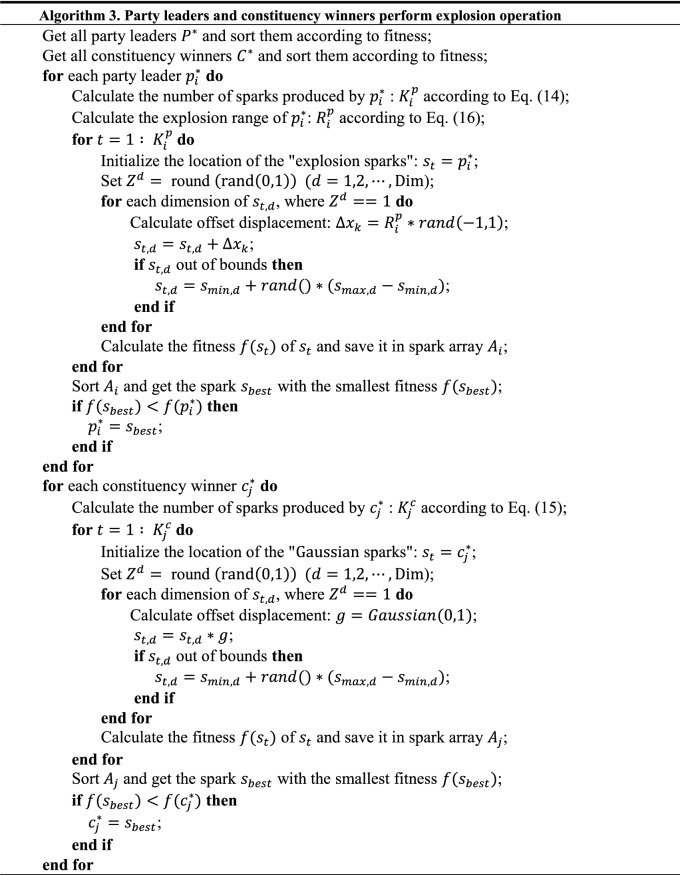


### Gaussian spark for verification of RPPUS

**Figure 5:**
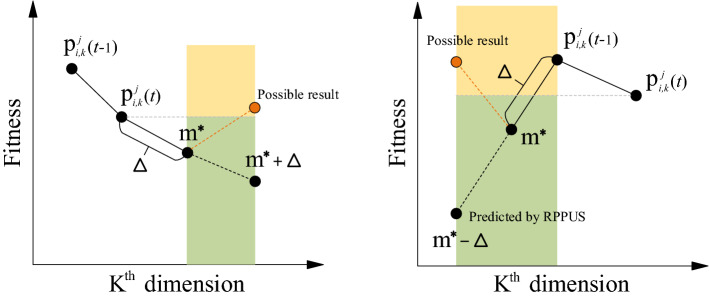
Potential flaws of RPPUS.

As mentioned earlier, RPPUS only predicts the favorable area where the search agent moves and lacks correctness verification after the update. In some cases, the fitness of the candidate solution after the update is worse than the fitness before the update. As shown in Fig. 5, RPPUS only roughly predicts based on three reference points. The green area is where we want the candidate solution to enter, but the candidate solution may enter the yellow area and cause the fitness to become worse. At this time, the candidate solution is regarded as a “problematic” solution and it should be corrected.

In this paper, the Gaussian spark in the FWA is used to correct the candidate solution whose fitness becomes worse after the update. The specific method is to generate three sparks around the candidate solution and judge whether there is a better solution than the candidate solution before the update among the three sparks, if there is, choose the best spark as the new candidate solution. If the fitness of all sparks is worse than that of the candidate solution before the update, the candidate solution before the update will be inherited and no change will be made. It should be noted that the Gaussian spark here is slightly different from the original firework algorithm because we stipulate that the number of sparks generated by the “problematic” solution is three. The pseudo-code of this process is shown in Algorithm 4.
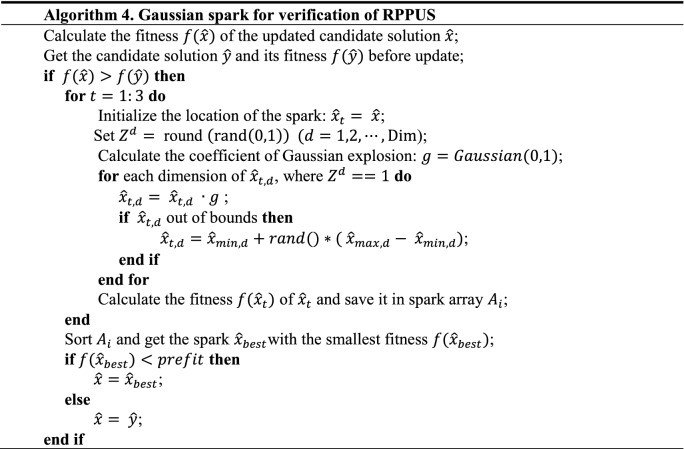


### Converged mobility center with bi-directional consideration

In PO, the party leader and the constituency winner are successively regarded as the center on which the position of the member is moved. If the two centers themselves are relatively close, it is not necessary to update twice. In response to this situation, we propose a new method to generate a new subgroup optimal solution as a mobile center—Converged Mobility Center with Bi-directional Consideration (CMC), which not only uses the advantages of both the party leader and the constituency winner but also maintains the population diversity.

**Figure 6:**
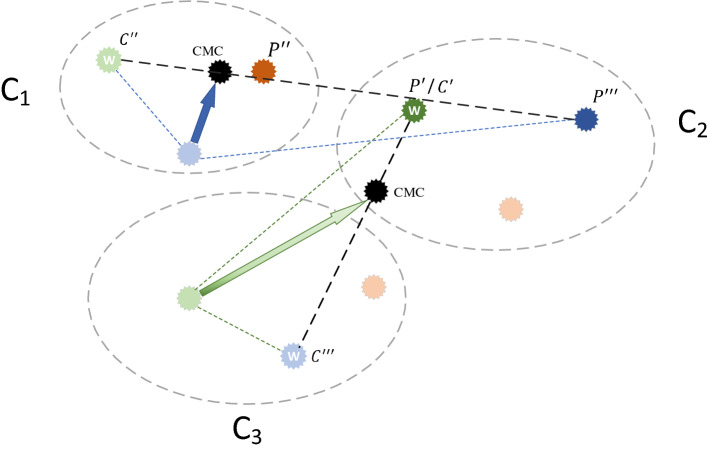
Converged Mobility Center with Bi-directional Consideration.

In order to improve their performance in the election, candidates not only refer to the advantages of party leaders but also compare and analyze with the constituency winners. This action should be carried out at the same time, not one after the other. The higher the ranking of the party leader of the candidate’s party among all party leaders, the more the candidate wants to be close to the party leader. In the same way, the better the constituency winner of the candidate's constituency ranks among all constituency winners, the candidate will prefer the constituency winner. CMC is proposed based on this consideration. As shown in Fig. 6, $P^{\prime}$ means ranking first among all party leaders, $P^{\prime\prime}$ means ranking second, $P^{\prime\prime\prime}$ means ranking third, and $C^{\prime}$ , $C^{\prime\prime}$ and $C^{\prime\prime\prime}$ indicate the ranking among the constituency winners. CMC will be generated near the higher-ranked party leader or constituency winner. The solution of CMC is shown in Eq. (17).

17 $$center_{i,j}^{k} = PF* p_{i,k}^{*} + CF* c_{j,k}^{*}$$

where PF represents the party weighting factor, CF represents the constituency weighting factor, $p_{i,k}^{*}$ indicates the value of the *k*th dimension of the party leader $p_{i}^{*}$, and $c_{j,k}^{*}$ indicates the value of the *k*th dimension of the party leader $c_{j}^{*}$.

The party weighting factor PF and the constituency weighting factor CF are calculated as follows:

18 $$PF = r_{1} *\frac{{N - PartyRank\left( {p_{i}^{*} } \right)}}{N},\quad PartyRank = sort(P^{*} )$$

19 $$CF = r_{2} *\frac{{N - {\text{Constituency}}Rank\left( {c_{j}^{*} } \right)}}{N},\quad ConstituencyRank = sort(C^{*} )$$

where $r_{1}$ and $r_{2}$ denotes the random value in the interval of [0, 1], $N$ indicates the total number of parties or constituencies.

### Computational complexity

Time complexity is a key criterion for judging the quality of an algorithm. To demonstrate the computational efficiency of GPOFWA, this section analyzes the computational complexity of PO and GPOFWA. The time complexity analysis of PO mainly includes three parts:

1. The time complexity of the population initialization phase is $O(ND)$, where $N$ represents the population size and $D$ represents denotes the dimensions variables of the problem.
2. The fitness value of each candidate is evaluated initially, and the time complexity is $O(NT_{obj} )$, where $T_{obj}$ denotes the cost of the objective function.
3. The main loop of the algorithm is the main time consumption. The time complexity of the election campaign stage is $O(2ND)$, $O(N)$ is the time complexity of party switching phase, $O(NT_{obj} )$ is the time complexity of the election stage, and the time complexity of parliamentary affairs stage is $O\left( {\sqrt N D} \right)$, and $T_{{{\text{max}}}}$ with each component is for the main loop. Therefore, the time complexity of the basic PO for $T_{{{\text{max}}}}$ loops can be computed as follows:
  $$O(PO) = O(ND) + O(NT_{obj} ) + T_{max} \times \left( {O(2ND) + O(N) + O(NT_{obj} ) + O\left( {\sqrt N D} \right)} \right)$$

In contrast, GPOFWA introduced the search strategy of the fireworks algorithm and adopted the Converged Mobility Center with bi-directional consideration. The time complexity of these two algorithms is different in the main loop. GPOFWA performs explosion spark and Gaussian explosion spark operations on party leaders and constituency winners to optimize subgroup optimal solutions. The time complexity of this process is $O\left( {2\sqrt N DK} \right)$, where $K$ represents the number of sparks generated by the subgroup optimal solution. Gaussian spark for verification of RPPUS and CMC are applied in the election campaign stage, the time complexity is $O(ND)$. Therefore, the time complexity of the GPOFWA for $T_{{{\text{max}}}}$ loops can be computed as follows:

$$O(GPOFWA) = O(ND) + O(NT_{obj} ) + T_{{{\text{max}}}} \times \left( {O(ND) + O(N) + O(NT_{obj} ) + O\left( {\sqrt N D} \right) + { }O\left( {2\sqrt N DK} \right)} \right)$$

We can conclude from the detailed analysis that they are of the same order of magnitude.

## Experiments and discussion

The performance of GPOFWA is evaluated on 30 basic benchmark functions in multiple dimensions (30 and 500), CEC2019 benchmark functions and three engineering optimization problems against a good combination of some advanced swarm intelligence algorithms. These test cases include various types (linear, nonlinear, and quadratic) of objective functions with the different number of decision variables and a range of types (linear inequalities, nonlinear equalities, and nonlinear inequalities), and the number of constraints. All simulation experiments are conducted on a computer with a Win10 operating system and Intel(R) Core (TM) i7-10750H GHz with 16 GB RAM. The proposed algorithm is coded in MATLAB R2020a.

### Comparison with other algorithms in low-dimensional functions

To verify the good performance of GPOFWA, we first used thirty benchmark functions for testing which are equally divided into two groups: unimodal function and multimodal function. The unimodal function (F1–F15) with the unique global optimal solution can reveal the exploitative capabilities of different algorithms, while the multimodal function (F16–F30) can be used to test the ability of the algorithm to avoid falling into the local optimal solution. It should be noted that the multimodal function test set also contains some fixed-dimensional functions, which show some optimization problems in the real world.

The detailed information of the unimodal function is shown in Table 1, including mathematical expressions, test dimensions, search ranges, and theoretical optimal values. The same details of multimodal functions are presented in Table 2. Moreover, in order to reflect the superiority of GPOFWA, we compare it with the existing advanced optimization algorithms, including HHO, GWO, SCA, SSA, WCA, WOA, LSA, and the original PO. The algorithms used for comparison and their parameter settings are all shown in Table 3. It is worth mentioning that parameter settings are based on the parameters used by the original author or the parameters widely used by various researchers. To ensure the fairness of the experiment, we compare the performance of the algorithms after running each experiment independently 30 times and the maximum number of objective function evaluations for all algorithms is set to 30,000.

**Table 1:** Detailed information of unimodal benchmark functions.

Function	Dim	Range	${F}_{min}$
${F}_{1}\left(x\right)={\sum }_{i=1}^{n}{x}_{i}^{2}$	30	[− 100, 100]	0
${F}_{2}\left(x\right)={\sum }_{i=1}^{n}i{x}_{i}^{4}+random\left[0, 1\right)$	30	[− 1.28, 1.28]	0
${F}_{3}\left(x\right)={\sum }_{i=1}^{n}{\left|{x}_{i}\right|}^{i+1}$	30	[− 1, 1]	0
${F}_{4}\left(x\right)={\sum }_{i=1}^{n}\left|{x}_{i}\right|$	30	[− 100, 100]	0
${F}_{5}\left(x\right)=\mathrm{max}\left(\left|{x}_{i}\right|,1\le i\le n\right)$	30	[− 100, 100]	0
${F}_{6}\left(x\right)=25+{\sum }_{i=1}^{n}\left(\lfloor{x}_{i}\rfloor\right)$	30	[− 5.12, 5.12]	25-6*n*
${F}_{7}\left(x\right)={\sum }_{i=1}^{n}{\left({\sum }_{j=1}^{i}{x}_{j}\right)}^{2}$	30	[− 100, 100]	0
${F}_{8}\left(x\right)={\sum }_{i=1}^{n}\left|{x}_{i}\right|+{\prod }_{i=1}^{n}\left|{x}_{i}\right|$	30	[− 100, 100]	0
${F}_{9}\left(x\right)={\sum }_{i=1}^{n}{x}_{i}^{10}$	30	[− 10, 10]	0
${F}_{10}\left(x\right)={\sum }_{i=1}^{n-1}{\left({x}_{i}^{2}\right)}^{\left({x}_{i+1}^{2}+1\right)}+{\left({x}_{i+1}^{2}\right)}^{{x}_{i}^{2}+1}$	30	[− 1, 4]	0
$${F}_{11}\left(x\right)=\begin{array}{c}{\sum }_{i=1}^\frac{D}{4}{\left({x}_{4i-3}+10{x}_{4i-2}\right)}^{2}+5{\left({x}_{4i-1}-{x}_{4i}\right)}^{2}+{\left({x}_{4i-2}-{x}_{4i-1}\right)}^{4}+10{\left({x}_{4i-3}-{x}_{4i}\right)}^{4}\end{array}$$	30	[− 4, 5]	0
${F}_{12}\left(x\right)={\sum }_{i=1}^{n}{x}_{i}^{2}+{\left(\sum_{i=1}^{n} 0.5i{x}_{i}\right)}^{2}+{\left(\sum_{i=1}^{n} 0.5i{x}_{i}\right)}^{4}$	30	[− 5, 10]	0
${F}_{13}\left(x\right)=\mathrm{exp}\left(-{\sum }_{i=1}^{n}{\left(\frac{{x}_{i}}{\beta }\right)}^{2m}\right) -2\mathrm{exp}\left(-{\sum }_{i=1}^{n}{x}_{i}^{2}\right){\prod }_{i=1}^{n}{\mathrm{cos}}^{2}\left({x}_{i}\right)$	30	[− 20, 20]	− 1
${F}_{14}\left(x\right)=2{x}_{1}^{2}-1.05{x}_{1}^{4}+\frac{{x}_{1}^{6}}{6}+{x}_{1}{x}_{2}+{x}_{2}^{2}$	2	[− 5, 5]	0
${F}_{15}\left(x\right)=0.26\left({x}_{1}^{2}+{x}_{2}^{2}\right)-0.48{x}_{1}{x}_{2}$	2	[− 10, 10]	0

**Table 2:** Detailed information of multimodal benchmark functions.

Function	Dim	Range	${F}_{min}$
${F}_{16}\left(x\right)=418.9829n-{\sum }_{i=1}^{n}-{x}_{i}\mathrm{sin}\left(\sqrt{\left|{x}_{i}\right|}\right)$	30	[− 500, 500]	0
${F}_{17}\left(x\right)={\sum }_{i=1}^{n}\left[{x}_{i}^{2}-10\mathrm{cos}\left(2\pi {x}_{i}\right)+10\right]$	30	[− 5.12, 5.12]	0
${F}_{18}\left(x\right)=1+{\sum }_{i=1}^{n}{\mathrm{sin}}^{2}\left({x}_{i}\right)-0.1{e}^{\left(\sum_{i=1}^{n} {x}_{i}^{2}\right)}$	30	[− 10, 10]	0.9
${F}_{19}\left(x\right)={\sum }_{i=1}^{n}\left|{x}_{i}\mathrm{sin}\left({x}_{i}\right)+0.1{x}_{i}\right|$	30	[− 10, 10]	0
${F}_{20}\left(x\right)={\sum }_{i=1}^{n}{\epsilon }_{i}{\left|{x}_{i}\right|}^{i}$	30	[− 5, 5]	0
${F}_{21}\left(x\right)=-20\mathrm{exp}\left(-0.2\sqrt{\frac{1}{n}\sum_{i=1}^{n} {x}_{i}^{2}}\right)-\mathrm{exp}\left(\frac{1}{n}\sum_{i=1}^{n} \mathrm{cos}\left(2\pi {x}_{i}\right)\right)+20+e$	30	[− 32, 32]	0
${F}_{22}\left(x\right)={\sum }_{i=1}^{n}8{\mathrm{sin}}^{2}\left[7{\left({x}_{i}-0.9\right)}^{2}\right]+6{\mathrm{sin}}^{2}\left[14{\left({x}_{1}-0.9\right)}^{2}\right]+{\left({x}_{i}-0.9\right)}^{2}$	30	[− 500, 500]	1
${F}_{23}\left(x\right)=1-\mathrm{cos}\left(2\pi \sqrt{\sum_{i=1}^{n} {x}_{i}^{2}}\right)+0.1\sqrt{\sum_{i=1}^{n} {x}_{i}^{2}}$	30	[− 100, 100]	0
${F}_{24}\left(x\right)=\frac{1}{2}{\sum }_{i=1}^{n}\left({x}_{i}^{4}-16{x}_{i}^{2}+5{x}_{i}\right)$	30	[− 5, 5]	− 39.16599 $\times n$
${F}_{25}\left(x\right)=1/4000{\sum }_{i=1}^{n}{x}_{i}^{2}-{\prod }_{i=1}^{n}\mathrm{cos}\left({x}_{i}/\sqrt{i}\right)+1$	30	[− 100, 100]	0
$${F}_{26}\left(x\right)=\begin{array}{c}\left(\sum_{i=1}^{n} {\mathrm{sin}}^{2}\left({x}_{i}\right)-{e}^{-{\Sigma }_{i=1}^{n}{x}_{i}^{2}}\right){e}^{-\sum_{i=1}^{n} {\mathrm{sin}}^{2}\sqrt{\left|{x}_{i}\right|}}\end{array}$$	30	[− 10, 10]	− 1
${F}_{27}\left(x\right)=\left(\sum_{i=1}^{n} \left|{x}_{i}\right|\right)\mathrm{exp}\left(-{\sum }_{i=1}^{n}\mathrm{sin}\left({x}_{i}^{2}\right)\right)$	30	[− 2$\pi$, 2$\pi$]	0
${F}_{28}\left(x\right)={x}^{2}+{y}^{2}+25\left({\mathrm{sin}}^{2}(x)+{\mathrm{sin}}^{2}(y)\right)$	2	[− 5, 5]	0
${F}_{29}\left(x\right)=-0.0001{\left(\left|\mathrm{sin}(x)\mathrm{sin}(y)\mathrm{exp}\left(\left|100-\frac{\sqrt{{x}^{2}+{y}^{2}}}{\pi }\right|\right)\right|+1\right)}^{0.1}$	2	[− 2, 2]	3
${F}_{30}\left(x\right)=-200{e}^{-0.2\sqrt{{x}^{2}+{y}^{2}}}+5{e}^{\mathrm{cos}(3x)+\mathrm{sin}(3y)}$	2	[− 32, 32]	− 195.629

**Table 3:** Parameter settings of the algorithm used for comparison.

Algorithm	Parameters	Value
GPOFWA	Number of parties/constituencies	8
	Lambda (initial party switching rate)	1
	Parameter $k$	50
	Parameter $R$	40
PO	Number of parties/constituencies	8
	Lambda (initial party switching rate)	1
HHO	Hawks numbers	42
GWO	Wolf numbers	42
	a (area vector)	[0, 2]
SCA	Solution numbers	42
	a (constant)	2
SSA	Salp numbers	42
WCA	Solution numbers	42
	Parameter $C$	2
	Parameter $\mu$	0.1
WOA	Whales number	42
	Parameter $A$	[0, 2]
	Parameter ${A}_{2}$	[0, 2]
LSA	Projectiles number	42
	Channel time	10

**Figure 7:**
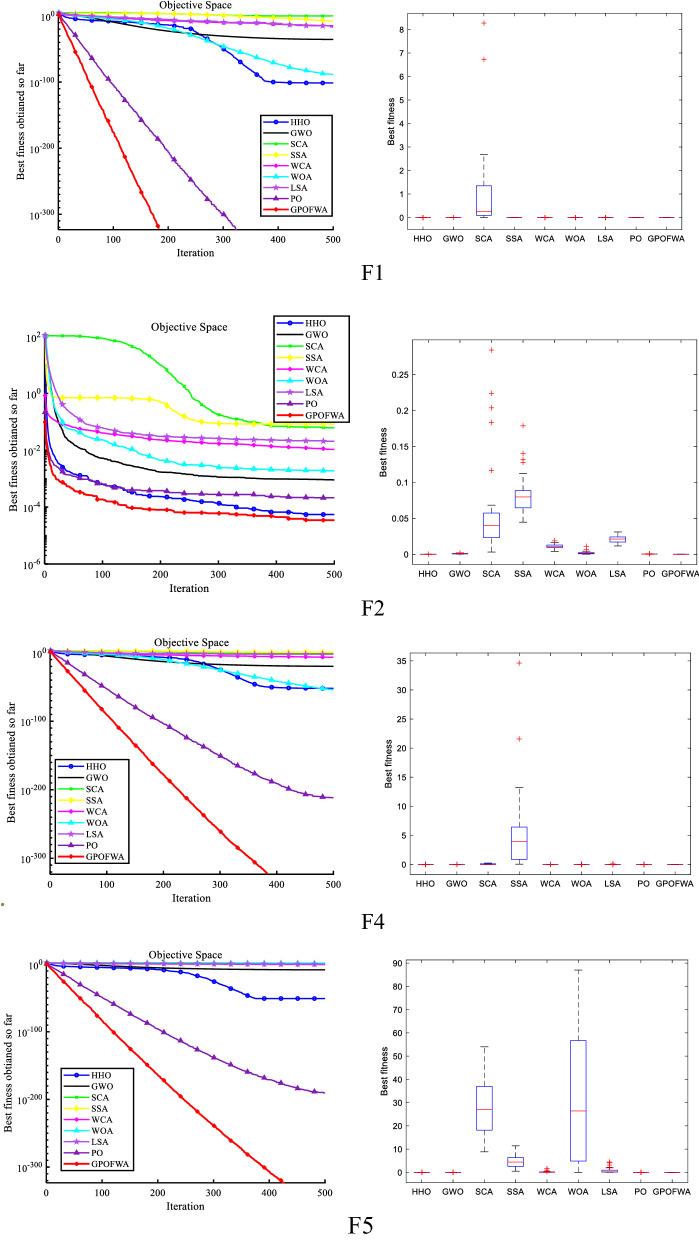

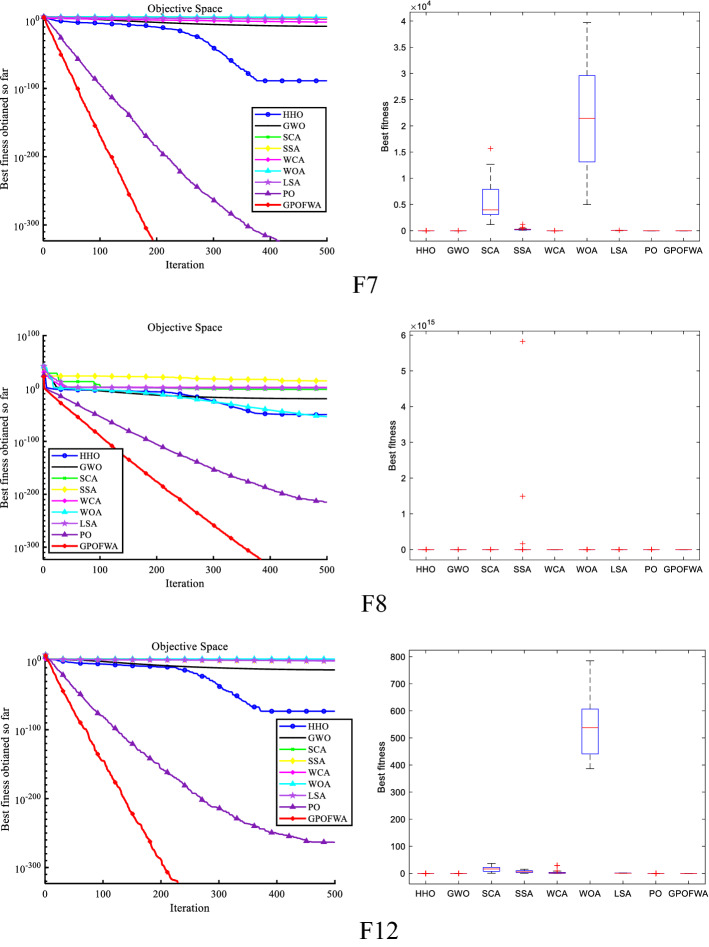
Qualitative results of some unimodal functions in 30 dimensions.

First of all, we tested the performance of all selected algorithms on F1–F15. And used three different statistics to start the first step of the evaluation. These statistics are the best fitness value (Best), the average fitness value (Mean), and the standard deviation (Std). Table 4 outlines the obtained results using these measures where the best ones are highlighted in bold text. It can be seen from the table that the proposed algorithm GPOFWA is superior to the original PO, and performs better than other advanced optimization algorithms. Especially for F4–F8 and F12, GPOFWA can find the theoretical optimal value of the function, while other algorithms are far different in terms of optimization accuracy. For the remaining unimodal functions, the performance of GPOFWA is also better than other algorithms. Not only does it converge faster, but it also achieves the best results in finding global optimal values. In order to reflect the superiority of GPOFWA in convergence speed, we also drew some convergence curves as shown in Fig. 7 based on the average fitness value of each generation in 30 experiments, and show the stability of the algorithm through the corresponding box plot. It can be seen from the figure that for most unimodal functions, GPOFWA can find the optimal value in a few iterations, which shows that its global optimization ability is stronger than other algorithms.

**Table 4:** Unimodal function test results of all selected algorithms.

${F}_{n}$	Stats	HHO	GWO	SCA	SSA	WCA	WOA	LSA	PO	GPOFWA
${F}_{1}$	Mean	3.27E−112	3.06E−36	0.852131	1.11E−08	7.12E−18	2.91E−92	5.24E−19	**0.0E+00**	**0.0E+00**
	Best	1.64E−113	1.63E−36	0.55677	1.02E−08	4.43E−19	7.07E−94	2.02E−19	**0.0E+00**	**0.0E+00**
	Std	4.39E−112	2.02E−36	0.417704	1.23E−09	9.44E−18	4.02E−92	4.55E−19	**0.0E+00**	**0.0E+00**
${F}_{2}$	Mean	4.15E−05	0.000649	0.02464	0.082534	0.006701	0.004491	0.021349	0.000171	**1.64E−05**
	Best	6.18E−05	0.0004	0.012528	0.049885	0.005301	0.000352	0.015262	4.48E−05	**1.07E−05**
	Std	4.99E−05	0.000352	0.017129	0.046173	0.001981	0.005854	0.008609	0.000179	**8.11E−06**
${F}_{3}$	Mean	9.61E−132	1.59E−127	5.57E−06	2.11E−07	7.20E−28	5.72E−143	3.96E−66	**0.0E+00**	**0.0E+00**
	Best	2.11E−138	6.77E−130	3.74E−07	1.67E−07	1.08E−32	1.60E−143	2.71E−69	**0.0E+00**	**0.0E+00**
	Std	1.36E−131	2.24E−127	7.35E−06	6.16E−08	1.02E−27	5.83E−143	5.60E−66	**0.0E+00**	**0.0E+00**
${F}_{4}$	Mean	1.40E−52	3.84E−09	24.5126	5.2992	0.137835	31.5447	0.507328	4.98E−192	**0.0E+00**
	Best	1.22E−59	7.25E−10	8.34198	1.1729	0.02232	0.005592	0.063907	9.23E−202	**0.0E+00**
	Std	7.17E−52	3.20E−09	11.3727	2.79292	0.135222	29.6858	0.379579	6.29E−200	**0.0E+00**
${F}_{5}$	Mean	0.000967	26.4269	4289.73	103.807	9.45568	27.2305	38.0473	4.80E−192	**0.0E+00**
	Best	0.000813	25.7264	246.468	75.9896	0.125462	27.1183	0.85238	6.20E−203	**0.0E+00**
	Std	0.000218	0.990773	5718.04	39.3403	13.1949	0.158699	52.6016	**0.0E+00**	**0.0E+00**
${F}_{6}$	Mean	2.99E−05	0.000983	0.050684	0.040825	0.012511	0.000726	0.019903	0.000258	**2.84E−05**
	Best	2.40E−05	0.000634	0.049177	0.021008	0.006514	0.00054	0.015872	0.000193	**2.99E−06**
	Std	8.26E−05	0.000494	0.002131	0.028026	0.00848	0.000264	0.005701	9.23E−05	**3.60E−05**
${F}_{7}$	Mean	1.62E−89	1.13E−09	5628.35	290.083	0.001705	21,356.7	21.1357	**0.0E+00**	**0.0E+00**
	Best	4.31E−113	1.74E−12	1159.88	100.405	0.000135	4970.73	2.89647	**0.0E+00**	**0.0E+00**
	Std	8.27E−89	1.84E−09	3643.7	208.889	0.003003	9330.73	13.258	**0.0E+00**	**0.0E+00**
${F}_{8}$	Mean	3.56E−50	4.14E−20	0.022274	2.49E+14	158.893	3.76E−54	7.72498	1.15E−215	**0.0E+00**
	Best	8.29E−60	3.17E−21	0.000181	216.132	1.44E−10	1.02E−59	1.72E−08	2.12E−227	**0.0E+00**
	Std	1.95E−49	5.35E−20	0.034437	1.09E+15	237.738	9.92E−54	20.9239	**0.0E+00**	**0.0E+00**
${F}_{9}$	Mean	**0.0E+00**	1.17E−119	138,063	2.02E−16	2.12E−47	1.15E−260	6.73E−57	**0.0E+00**	**0.0E+00**
	Best	**0.0E+00**	3.01E−131	2.39E−05	5.90E−34	8.58E−66	**0.0E+00**	1.15E−62	**0.0E+00**	**0.0E+00**
	Std	**0.0E+00**	6.01E−119	573,729	7.72E−16	1.09E−46	**0.0E+00**	1.95E−56	**0.0E+00**	**0.0E+00**
${F}_{10}$	Mean	5.12E−104	7.67E−39	0.000546	3.86E−11	8.27E−19	2.96E−92	1.31E−15	**0.0E+00**	**0.0E+00**
	Best	9.89E−124	2.32E−40	1.73E−06	1.88E−11	1.53E−24	2.25E−101	1.16E−22	**0.0E+00**	**0.0E+00**
	Std	2.78E−103	1.18E−38	0.000752	1.17E−11	2.87E−18	1.56E−91	6.95E−15	**0.0E+00**	**0.0E+00**
${F}_{11}$	Mean	3.45E−103	1.00E−05	4.13447	1.51361	2.81E−05	2.08E−06	0.007704	**0.0E+00**	**0.0E+00**
	Best	2.55E−119	8.83E−07	0.004147	0.271445	7.52E−06	8.41E−93	0.000348	**0.0E+00**	**0.0E+00**
	Std	1.72E−102	8.82E−06	9.23443	1.26208	2.06E−05	5.89E−06	0.031663	**0.0E+00**	**0.0E+00**
${F}_{12}$	Mean	6.73E−74	8.87E−14	16.2	6.19244	4.07989	539.19	0.685463	2.83E−264	**0.0E+00**
	Best	1.77E−97	5.87E−16	0.609656	0.541016	0.006203	386.573	0.08349	1.39E−314	**0.0E+00**
	Std	3.19E−73	1.42E−13	9.63937	4.27787	7.39854	105.514	0.454347	**0.0E+00**	**0.0E+00**
${F}_{13}$	Mean	**−** **1.0E+00**	5.05E−140	3.91E−187	6.18E−182	4.34E−232	**−** 0.6	6.87E−136	**−** 0.23333	**−** **1.0E+00**
	Best	**−** **1.0E+00**	3.46E−194	1.02E−204	3.56E−202	4.34E−232	**−** **1.0E+00**	1.20E−192	**−** **1.0E+00**	**−** **1.0E+00**
	Std	**0.0E+00**	2.76E−139	**0.0E+00**	**0.0E+00**	**0.0E+00**	0.498273	3.76E−135	0.430183	**0.0E+00**
${F}_{14}$	Mean	1.44E−116	1.04E−260	2.22E−80	4.56E−15	5.50E−40	2.72E−103	1.25E−261	**0.0E+00**	**0.0E+00**
	Best	2.71E−134	4.23E−321	1.41E−92	7.52E−17	7.86E−46	3.46E−125	1.12E−269	**0.0E+00**	**0.0E+00**
	Std	7.89E−116	**0.0E+00**	7.99E−80	5.48E−15	8.28E−40	1.49E−102	**0.0E+00**	**0.0E+00**	**0.0E+00**
${F}_{15}$	Mean	1.08E−136	2.28E−160	2.75E−66	8.87E−16	2.20E−40	4.98E−247	1.71E−159	**0.0E+00**	**0.0E+00**
	Best	9.19E−180	3.49E−181	3.70E−80	5.20E−18	1.23E−43	4.84E−277	1.33E−178	**0.0E+00**	**0.0E+00**
	Std	4.43E−136	9.15E−160	1.44E−65	1.22E−15	5.55E−40	**0.0E+00**	8.04E−159	**0.0E+00**	**0.0E+00**

Significant values are in bold.

**Figure 8:**
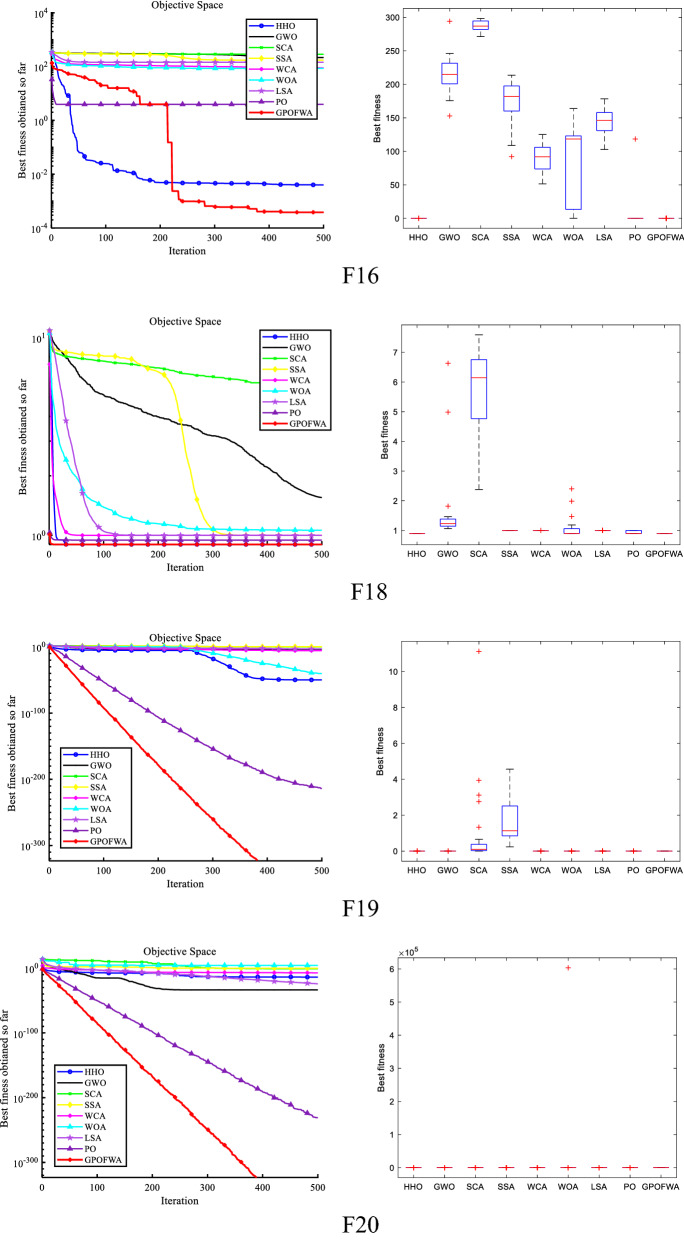

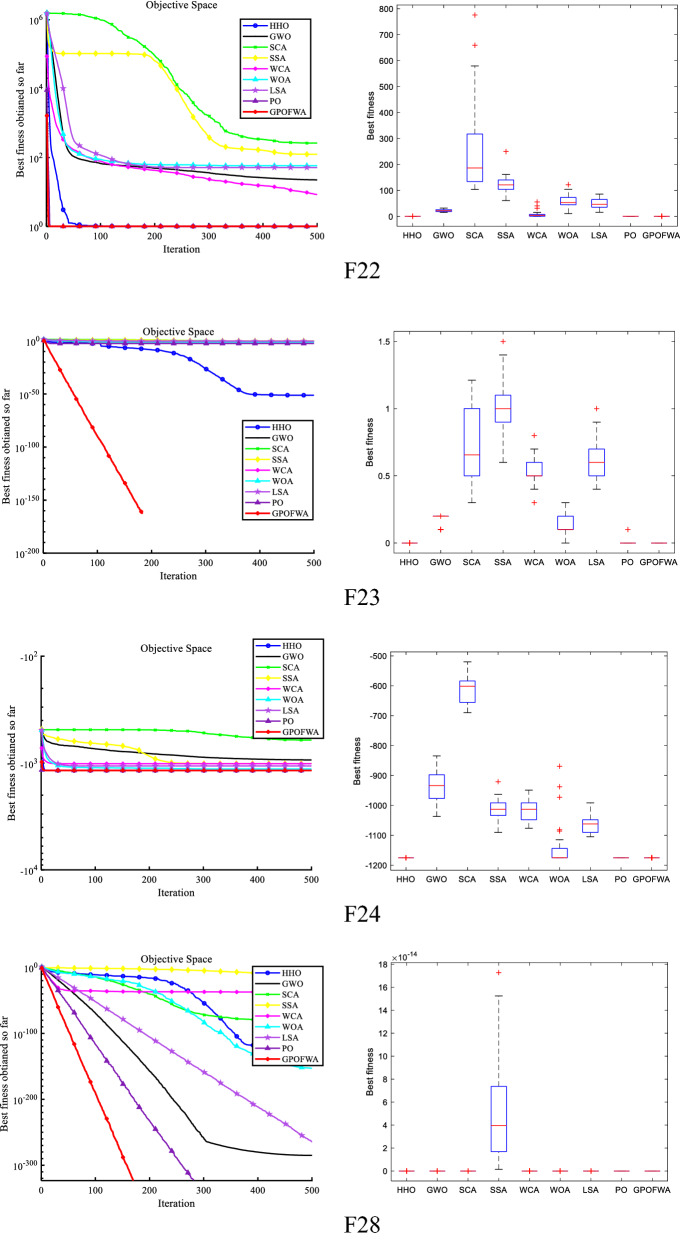
Qualitative results of some multimodal functions in 30 dimensions.

By testing the unimodal function F1–F15, we can find the powerful exploitative capability of GPOFWA. To evaluate the exploration capability of GPOFWA, we used the multimodal function set F16–F30 for testing. As with the unimodal function test, we also use the best fitness value (Best), the average fitness value (Mean), and the standard deviation (Std) three statistics to illustrate the experimental results. The experimental results are shown in Table 5. It can be seen from the table that GPOFWA performs better on the multidimensional function test set than other advanced optimization algorithms. For example, in functions such as F16–F20 and F23, GPOFWA has higher optimization accuracy than other optimization algorithms. Secondly, we can find that the variance corresponding to the running results of GPOFWA is very small, most of which are 0 or close to 0, which means that GPOFWA is relatively stable in 30 runs. In addition, we also drew the convergence curve as shown in Fig. 8 based on the results of 30 runs and show the stability of the algorithm through the corresponding box plot. It can be seen from the figure that the convergence speed and optimization accuracy of GPOFWA are superior. Considering the performance of GPOFWA on the unimodal function and multimodal function test sets, we can find that GPOFWA not only has good exploitation capability but also performs well in exploration capability.

**Table 5:** Multimodal function test results of all selected algorithms.

${F}_{n}$	Stats	HHO	GWO	SCA	SSA	WCA	WOA	LSA	PO	GPOFWA
${F}_{16}$	Mean	0.00392	234.851	291.308	165.807	85.3049	65.1877	155.304	59.2192	**0.000155**
	Best	0.003725	226.485	281.702	161.268	75.7705	0.054689	134.24	1.27E−05	**4.58E−05**
	Std	0.000275	11.8308	13.5841	6.4189	13.4837	92.1121	29.7894	83.7485	**0.000154**
${F}_{17}$	Mean	**0.0E+00**	1.15682	28.1853	39.3008	42.7932	1.89E−15	62.9476	0.994959	**0.0E+00**
	Best	**0.0E+00**	**0.0E+00**	0.006467	13.9294	16.9143	**0.0E+00**	42.7832	**0.0E+00**	**0.0E+00**
	Std	**0.0E+00**	2.59919	27.8158	16.2666	14.015	1.04E−14	16.7692	5.44962	**0.0E+00**
${F}_{18}$	Mean	**0.9E+00**	1.49474	5.86083	1.0E+00	1.0E+00	1.07092	1.0E+00	0.933333	**0.9E+00**
	Best	**0.9E+00**	1.03939	2.53771	1.0E+00	1.0E+00	**0.9E+00**	1.0E+00	**0.9E+00**	**0.9E+00**
	Std	**4.52E−16**	0.853118	1.2096	3.01E−11	6.75E−16	0.228393	1.66E−11	0.047946	**4.52E−16**
${F}_{19}$	Mean	3.51E−55	2.26E−19	0.061249	0.809466	1.43E−09	6.65E−58	7.81E−06	1.57E−202	**0.0E+00**
	Best	9.24E−61	8.44E−21	0.036909	0.674069	5.28E−10	1.92E−58	2.26E−06	2.38E−221	**0.0E+00**
	Std	4.97E−55	3.08E−19	0.034423	0.191481	1.28E−09	6.69E−58	7.85E−06	**0.0E+00**	**0.0E+00**
${F}_{20}$	Mean	1.35E−14	3.10E−34	0.109068	0.67503	4.75E−08	20,117.7	5.81E−25	1.32E−231	**0.0E+00**
	Best	4.50E−73	2.38E−54	3.88E−06	0.00029	1.28E−10	5.79E−30	4.64E−32	2.38E−248	**0.0E+00**
	Std	7.42E−14	1.65E−33	0.306688	1.25076	1.20E−07	110,189	1.57E−24	**0.0E+00**	**0.0E+00**
${F}_{21}$	Mean	**−** **8.88E−16**	3.55E−14	10.9147	2.03485	0.000415	3.02E−15	1.75891	**−** **8.88E−16**	**−** **8.88E−16**
	Best	**−** **8.88E−16**	2.75E−14	0.014726	0.931305	4.26E−12	**−** **8.88E−16**	8.16E−11	**−** **8.88E−16**	**−** **8.88E−16**
	Std	**0.0E+00**	3.81E−15	9.82257	0.605984	0.001602	1.43E−15	1.16894	**0.0E+00**	**0.0E+00**
${F}_{22}$	Mean	1.00022	22.4015	261.62	123.734	8.35551	57.9046	51.2548	**1.0E+00**	**1.0E+00**
	Best	**1.0E+00**	15.5343	104.113	61.313	**1.0E+00**	11.4593	16.2528	**1.0E+00**	**1.0E+00**
	Std	0.00028	4.58991	180.526	35.0562	12.7251	24.5662	20.2151	**0.0E+00**	**0.0E+00**
${F}_{23}$	Mean	9.88E−55	0.199873	0.629963	0.999873	0.549873	0.199873	0.499873	1.69E−202	**0.0E+00**
	Best	1.71E−56	0.199873	0.601173	0.799873	0.499873	0.099873	0.499873	2.48E−221	**0.0E+00**
	Std	1.37E−54	1.91E−11	0.040715	0.282843	0.070711	0.141421	2.34E−08	1.57E−223	**0.0E+00**
${F}_{24}$	Mean	**−** **1174.98**	**−** 935.519	**−** 610.207	**−** 1011.47	**−** 1015.24	**−** 1135.68	**−** 1065.66	**−** **1174.98**	**−** **1174.98**
	Best	**−** **1174.98**	**−** 1036.94	**−** 689.914	**−** 1090.16	**−** 1076.03	**−** 1174.96	**−** 1104.3	**−** **1174.98**	**−** **1174.98**
	Std	0.002945	50.8735	42.3432	37.9842	36.2134	76.7754	31.2673	2.31E−13	**4.59E−15**
${F}_{25}$	Mean	**0.0E+00**	0.001342	0.35926	0.010515	0.012955	0.001388	0.025707	**0.0E+00**	**0.0E+00**
	Best	**0.0E+00**	**0.0E+00**	0.000214	1.49E−08	**0.0E+00**	**0.0E+00**	**0.0E+00**	**0.0E+00**	**0.0E+00**
	Std	**0.0E+00**	0.004322	0.259761	0.00864	0.015486	0.007602	0.028341	**0.0E+00**	**0.0E+00**
${F}_{26}$	Mean	**−** **1.0E+00**	4.51E−16	1.84E−10	6.20E−23	2.51E−30	**−** 0.16667	1.89E−22	**−** 0.86667	**−** **1.0E+00**
	Best	**−** **1.0E+00**	2.17E−16	6.27E−11	2.93E−23	1.90E−37	**−** **1.0E+00**	3.42E−34	**−** **1.0E+00**	**−** **1.0E+00**
	Std	**−** **1.0E+00**	1.52E−16	1.10E−10	3.38E−23	9.31E−30	0.379049	1.04E−21	0.345746	**−** **1.0E+00**
${F}_{27}$	Mean	**3.51E−12**	1.72E−08	4.51E−10	3.46E−11	1.73E−11	3.69E−12	5.99E−12	**3.51E−12**	**3.51E−12**
	Best	**3.51E−12**	1.44E−11	9.00E−11	1.52E−11	1.64E−11	**3.51E−12**	4.43E−12	**3.51E−12**	**3.51E−12**
	Std	5.58E−15	3.43E−08	2.34E−10	2.52E−11	3.16E−13	3.39E−13	8.78E−13	4.33E−27	**5.61E−30**
${F}_{28}$	Mean	5.52E−120	8.19E−286	4.22E−81	5.16E−14	5.73E−38	1.71E−153	2.65E−265	**0.0E+00**	**0.0E+00**
	Best	9.47E−138	**0.0E+00**	2.15E−91	1.52E−15	1.33E−47	2.26E−183	5.65E−272	**0.0E+00**	**0.0E+00**
	Std	2.08E−119	**0.0E+00**	1.38E−80	4.56E−14	2.29E−37	6.53E−153	**0.0E+00**	**0.0E+00**	**0.0E+00**
${F}_{29}$	Mean	**3.0E+00**	3.00001	3.00003	**3.0E+00**	**3.0E+00**	3.00001	**3.0E+00**	7.24095	**3.0E+00**
	Best	**3.0E+00**	**3.0E+00**	**3.0E+00**	**3.0E+00**	**3.0E+00**	**3.0E+00**	**3.0E+00**	**3.0E+00**	**3.0E+00**
	Std	3.51E−08	7.58E−06	6.02E−05	9.09E−14	1.68E−15	1.44E−05	1.02E−15	7.55564	**4.82E−09**
${F}_{30}$	Mean	**−** **195.629**	**−** **195.629**	**−** **195.629**	**−** **195.629**	**−** **195.629**	**−** **195.629**	**−** **195.629**	**−** **195.629**	**−** **195.629**
	Best	**−** **195.629**	**−** **195.629**	**−** **195.629**	**−** **195.629**	**−** **195.629**	**−** **195.629**	**−** **195.629**	**−** **195.629**	**−** **195.629**
	Std	7.05E−09	2.93E−08	9.87E−05	7.90E−13	5.78E−14	2.63E−08	5.78E−14	5.78E−14	**7.10E−15**

Significant values are in bold.

### Comparison with other algorithms in high-dimensional functions

**Figure 9:**
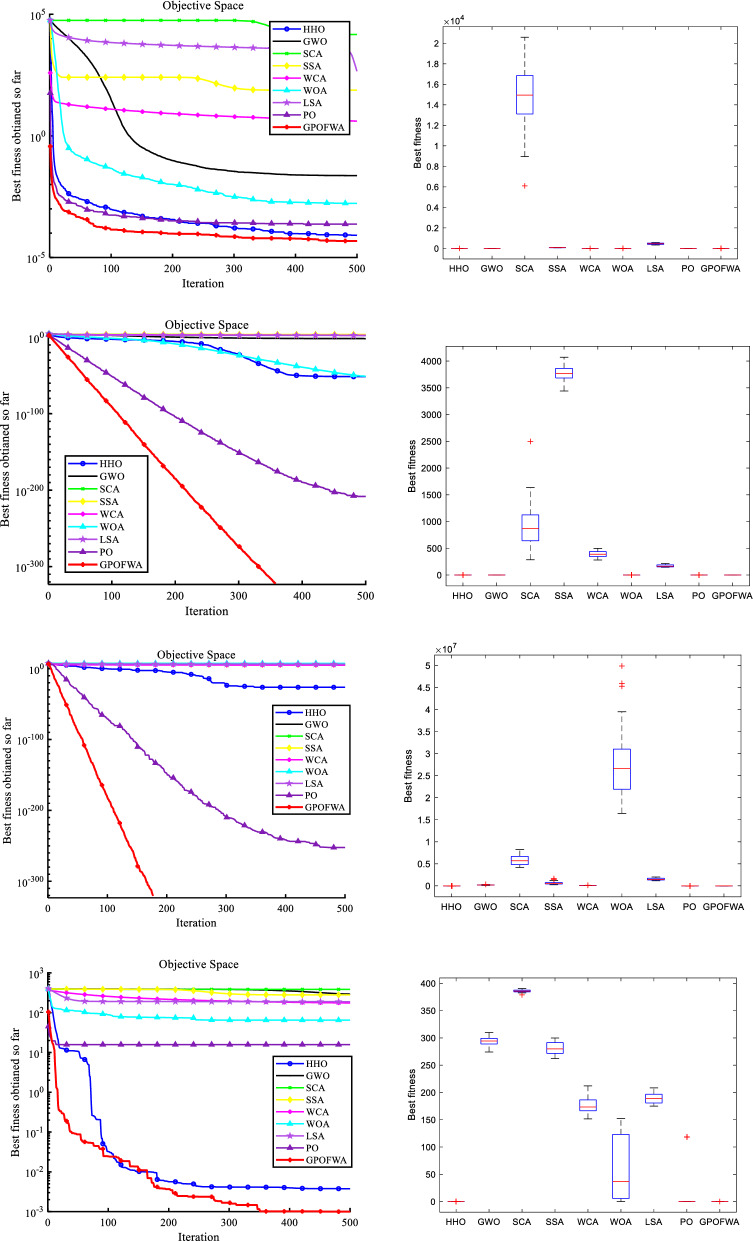

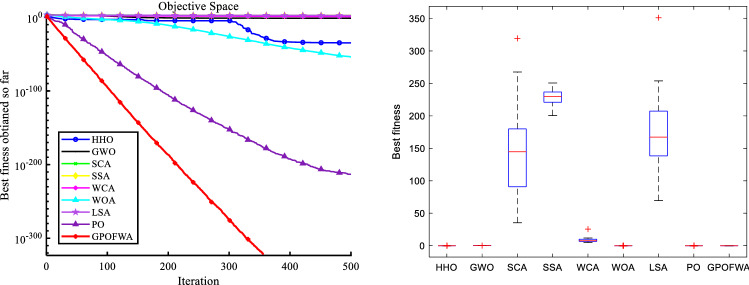
Qualitative results of F2, F4, F8, F16 and F20 functions in 500 dimensions.

To test the performance of the GPOFWA algorithm on high-dimensional problems, we tested unimodal and multimodal functions of 500 dimensions. It should be noted that the test function used in 4.1 contains some fixed dimension functions, so we chose F1–F10, F16–F25 for testing. For each function, the parameters are the same as those mentioned above. Figure 9 shows the qualitative analysis of functions in 500 dimensions. We also use the best fitness value (Best), the average fitness value (Mean), and the standard deviation (Std) three statistics to illustrate the experimental results. The experimental results are shown in Table 6. Similar to the low-dimensional case, GPOFWA also exhibits superior performance in high-dimensional functions. As shown in Fig. 9, it can be clearly seen that for unimodal functions such as F2, F4, and F8, GPOFWA has faster convergence speed and higher convergence accuracy, while for multimodal functions such as F16, GPOFWA shows its ability to avoid local optimal. From the results, the scalability of the proposed algorithm in terms of the number of variables of the optimization problem can be seen.

**Table 6:** Results of unimodal and multimodal functions in 500 dimensions.

${F}_{n}$	Stats	HHO	GWO	SCA	SSA	WCA	WOA	LSA	PO	GPOFWA
${F}_{1}$	Mean	1.38E−101	6.70E−05	192,795	46,116.9	782.025	1.02E−83	58.0799	**0.0E+00**	**0.0E+00**
	Best	9.26E−119	2.67E−05	70,773	41,245.9	451.696	7.84E−96	52.0459	**0.0E+00**	**0.0E+00**
	Std	5.52E−101	1.98E−05	72,415.1	3117.24	224.549	5.60E−83	2.54923	**0.0E+00**	**0.0E+00**
${F}_{2}$	Mean	8.11E−05	0.023221	14,699.5	75.9363	4.04729	0.001671	455.103	0.000236	**4.78E−05**
	Best	3.93E−06	0.012287	6096.39	59.572	2.52734	0.000105	327.357	4.53E−06	**1.84E−06**
	Std	9.41E−05	0.005217	3276.52	10.3166	0.553167	0.002025	65.1496	0.000186	**4.91E−05**
${F}_{3}$	Mean	2.95E−134	2.77E−07	1.60909	4.73E−07	1.01E−14	1.46E−137	2.57E−09	**0.0E+00**	**0.0E+00**
	Best	3.95E−148	1.30E−19	0.553188	1.35E−08	3.43E−17	1.97E−158	2.97E−12	**0.0E+00**	**0.0E+00**
	Std	7.53E−134	9.94E−07	0.399443	6.38E−07	3.57E−14	5.41E−137	4.57E−09	**0.0E+00**	**0.0E+00**
${F}_{4}$	Mean	3.41E−52	0.017427	938.128	3763.77	390.817	6.89E−52	169.327	5.23E−209	**0.0E+00**
	Best	2.99E−60	0.01276	284.762	3440.05	279.354	1.45E−60	145.075	4.31E−224	**0.0E+00**
	Std	1.35E−51	0.002987	443.239	150.906	55.9311	2.82E−51	21.9515	**0.0E+00**	**0.0E+00**
${F}_{5}$	Mean	5.22E−52	55.7736	98.7412	29.3284	23.5218	75.3936	80.9725	8.36E−182	**0.0E+00**
	Best	1.09E−59	43.3828	97.2443	25.3468	19.6904	0.056031	77.3959	1.04E−201	**0.0E+00**
	Std	2.34E−51	5.22121	0.455195	2.09579	1.82314	25.3531	1.63515	**0.0E+00**	**0.0E+00**
${F}_{6}$	Mean	**−** **2975**	**−** 1094.53	**−** 735.067	**−** 1178.23	**−** 2795.37	**−** **2975**	**−** 1455.63	**−** **2975**	**−** **2975**
	Best	**−** **2975**	**−** 1171	**−** 866	**−** 1333	**−** 2935	**−** **2975**	**−** 1564	**−** **2975**	**−** **2975**
	Std	**0.0E+00**	39.4136	38.9145	90.3311	74.0636	**0.0E+00**	63.9722	**0.0E+00**	**0.0E+00**
${F}_{7}$	Mean	0.000441	84.9981	180,891	45,500.9	836.426	13.1285	58.2665	**0.0E+00**	**0.0E+00**
	Best	5.99E−07	79.7633	33,046.2	40,400.4	546.971	7.28153	53.6144	**0.0E+00**	**0.0E+00**
	Std	0.000498	2.06337	74,563	3087.91	183.717	2.97662	1.9892	**0.0E+00**	**0.0E+00**
${F}_{8}$	Mean	4.91E−27	193,425	5.81E+06	615,504	79,634.2	2.84E+07	1.53E+06	4.49E−253	**0.0E+00**
	Best	1.60E−90	82,263.3	4.17E+06	245,354	53,562.8	1.65E+07	1.16E+06	1.04E−254	**0.0E+00**
	Std	2.69E−26	57,070.1	1.11E+06	341,055	15,655.1	8.59E+06	223,555	1.25E−201	**0.0E+00**
${F}_{9}$	Mean	7.97E−53	3.40E−20	0.035994	2.03E+13	87.5152	1.20E−54	1.8042	4.07E−218	**0.0E+00**
	Best	2.24E−61	5.00E−21	0.000528	257.593	1.67E−10	1.38E−62	3.16E−08	5.18E−227	**0.0E+00**
	Std	4.12E−52	2.51E−20	0.051	1.09E+14	205.512	3.04E−54	5.10198	**0.0E+00**	**0.0E+00**
${F}_{10}$	Mean	**0.0E+00**	1.68E−11	1.76E+11	121,177	6.90548	1.86E−245	2.34E−05	**0.0E+00**	**0.0E+00**
	Best	**0.0E+00**	1.73E−17	1.42E+11	35,850.9	0.554854	4.18E−320	2.79E−06	**0.0E+00**	**0.0E+00**
	Std	**0.0E+00**	6.55E−11	1.79E+10	65,964.2	11.007	**0.0E+00**	2.14E−05	**0.0E+00**	**0.0E+00**
${F}_{16}$	Mean	0.003771	293.832	385.996	280.997	175.47	64.8972	189.028	15.7918	**0.000998**
	Best	2.24E−05	274.155	378.749	262.217	151.633	0.157407	175.006	1.35E−05	**1.27E−05**
	Std	0.005274	9.07825	2.36353	11.1739	14.3605	59.507	9.79229	40.9496	**0.001788**
${F}_{17}$	Mean	**0.0E+00**	46.8127	1183.56	2609.97	773.186	3.03E−14	2283.1	**0.0E+00**	**0.0E+00**
	Best	**0.0E+00**	22.2264	455.69	2427.45	548.655	**0.0E+00**	1933.33	**0.0E+00**	**0.0E+00**
	Std	**0.0E+00**	18.2103	429.877	108.618	127.924	1.66E−13	146.664	**0.0E+00**	**0.0E+00**
${F}_{18}$	Mean	**0.9E+00**	14.3207	173.587	90.9618	2.27417	**0.9E+00**	4.35765	0.973333	**0.9E+00**
	Best	**0.9E+00**	4.0936	167.937	84.4045	1.16922	**0.9E+00**	3.89337	**0.9E+00**	**0.9E+00**
	Std	4.52E−16	37.6994	2.07128	3.70901	1.42388	4.97E−16	0.266574	0.044978	**3.51E−16**
${F}_{19}$	Mean	2.06E−35	0.044025	144.858	228.849	8.67805	1.15E−54	173.972	8.34E−214	**0.0E+00**
	Best	4.73E−63	0.03166	35.3217	200.308	4.9412	2.33E−60	69.4252	**0.0E+00**	**0.0E+00**
	Std	1.13E−34	0.007583	69.8559	13.3428	3.7008	3.23E−54	59.0763	**0.0E+00**	**0.0E+00**
${F}_{20}$	Mean	3.42E−14	3.87E−35	0.08697	2.13557	0.000683	0.001337	1.69E−25	9.06E−232	**0.0E+00**
	Best	1.68E−60	8.98E−59	6.10E−06	0.000428	3.16E−10	8.27E−47	4.32E−34	9.62E−249	**0.0E+00**
	Std	1.87E−13	1.86E−34	0.200318	8.80287	0.00351	0.004847	5.35E−25	**0.0E+00**	**0.0E+00**
${F}_{21}$	Mean	**−** **8.88E−16**	0.000377	19.281	11.956	7.05866	2.19E−15	17.4668	**−** **8.88E−16**	**−** **8.88E−16**
	Best	**−** **8.88E−16**	0.00026	7.60267	11.3273	4.82403	**−** **8.88E−16**	16.5547	**−** **8.88E−16**	**−** **8.88E−16**
	Std	**0.0E+00**	7.57E−05	3.53528	0.270896	4.38871	2.59E−15	0.406831	**0.0E+00**	**0.0E+00**
${F}_{22}$	Mean	1.0071	1544.27	4.37E+06	1.21E+06	49,205.5	1103.26	3543.23	**1.0E+00**	**1.0E+00**
	Best	1.00002	1366.25	1.24E+06	1.01E+06	29,072.8	857.55	3070.82	**1.0E+00**	**1.0E+00**
	Std	0.00978	153.919	1.92E+06	75,423.3	12,739.5	187.365	325.554	**0.0E+00**	1.13E−05
${F}_{23}$	Mean	1.01E−51	0.833207	43.8876	27.3299	14.3978	0.126553	6.94915	**0.0E+00**	**0.0E+00**
	Best	3.12E−61	0.699873	28.8171	25.8999	11.9999	1.43E−45	3.70026	**0.0E+00**	**0.0E+00**
	Std	5.14E−51	0.06609	8.62566	0.740061	1.18148	0.069125	2.46732	**0.0E+00**	**0.0E+00**
${F}_{24}$	Mean	**−** **1174.98**	**−** 935.519	**−** 610.207	**−** 1011.47	**−** 1015.24	**−** 1135.68	**−** 1065.66	**−** **1174.98**	**−** **1174.98**
	Best	**−** **1174.98**	**−** 1036.94	**−** 689.914	**−** 1090.16	**−** 1076.03	**−** 1174.96	**−** 1104.3	**−** **1174.98**	**−** **1174.98**
	Std	0.002945	50.8735	42.3432	37.9842	36.2134	76.7754	31.2673	2.31E−13	**4.59E−15**
${F}_{25}$	Mean	**0.0E+00**	0.002372	50.611	12.4213	1.13144	**0.0E+00**	0.292623	**0.0E+00**	**0.0E+00**
	Best	**0.0E+00**	8.31E−08	18.5269	10.7749	0.892625	**0.0E+00**	0.237605	**0.0E+00**	**0.0E+00**
	Std	**0.0E+00**	0.009047	18.0174	0.859874	0.089444	**0.0E+00**	0.029358	**0.0E+00**	**0.0E+00**

Significant values are in bold.

### Comparison with other algorithms on CEC2019 benchmark functions

**Figure 10:**
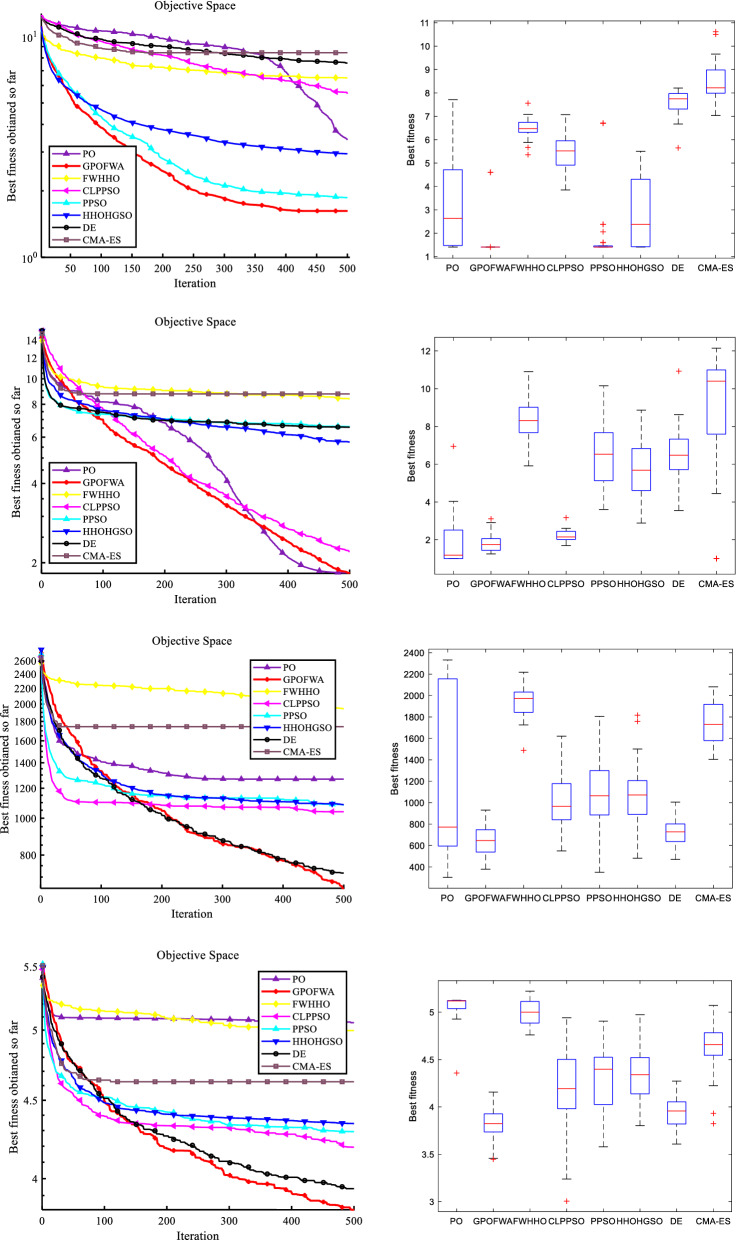
Qualitative results of F3, F6, F7 and F8 in CEC2019 benchmark functions.

By testing 30 classic benchmark functions in low and high dimensions, we can already find the excellent performance of GPOFWA. To further explore the effectiveness of the proposed method, we also use the CEC2019 benchmark function for testing. The CEC2019 benchmark function contains a number of shifted rotated functions to test the stability of the algorithm against function shifts. It is worth mentioning that the comparison algorithms we use in this section are some advanced and hybrid algorithms, not the basic algorithm used above. These algorithms are FWHHO^39^, PPSO^40^, CLPPSO^40^, HHOHGSO^41^, DE^15^ and CMA-ES^42^. The algorithms used for comparison and their parameter settings are based on the parameters used by the original author or the parameters widely used by various researchers. To ensure the fairness of the experiment, we compare the performance of the algorithms after running each experiment independently 30 times. Figure 10 shows a qualitative analysis of some CEC2019 benchmark functions and Table 7 shows results of CEC2019 benchmark functions. From the experimental results, GPOFWA can achieve better scores on F3, F6, F7, F8 of CEC2019, and it can be seen from the box plot that GPOFWA is more stable than other algorithms. Although not optimal on other functions, the results obtained using GPOFWA can be as close to optimal as possible.

**Table 7:** Results of CEC2019 benchmark functions.

${F}_{n}$	Stats	PO	FWHHO	PPSO	CLPPSO	HHOHGSO	DE	CMA-ES	GPOFWA
	Mean	**1.0E+00**	90.1385	9152.03	3.01E+06	**1.0E+00**	4.36E+06	6.86E+06	**1.0E+00**
${F}_{1}$	Best	**1.0E+00**	**1.0E+00**	**1.0E+00**	67,550	**1.0E+00**	1.04E+06	23,668.3	**1.0E+00**
	Std	**0.0E+00**	209.967	28,810.4	1.87E+06	**0.0E+00**	2.18E+06	2.20E+07	**0.0E+00**
	Mean	5.0E+00	36.6877	77.2991	2557.8	**4.93033**	3565.89	7593.51	5.0E+00
${F}_{2}$	Best	5.0E+00	4.28317	4.78953	1264.85	**4.24097**	1660.37	1711.56	5.0E+00
	Std	**7.22E−15**	98.8404	121.05	671.586	0.213082	571.946	4715.88	6.31E−12
	Mean	3.40324	6.49062	1.86345	5.50316	2.9452	7.57566	8.44484	**1.6224**
${F}_{3}$	Best	1.40916	5.35713	**1.40913**	3.84899	1.40917	5.64986	7.03099	**1.40913**
	Std	2.00073	0.811524	1.3427	0.80288	1.42588	0.549887	0.892922	**0.45333**
	Mean	**10.4785**	73.6007	41.5939	13.3899	35.859	15.6882	66.7327	41.0633
${F}_{4}$	Best	**5.9748**	58.3051	22.8891	9.46962	15.0433	10.867	46.0116	19.9042
	Std	3.64122	5.4947	13.3308	2.14732	8.37975	**2.77638**	8.80164	13.522
	Mean	**1.01822**	19.6206	1.61928	1.15534	1.54582	1.16497	5.87262	1.55077
${F}_{5}$	Best	**1.0E+00**	8.2182	1.16243	1.07674	1.14981	1.08251	**1.0E+00**	**1.0E+00**
	Std	**0.01086**	8.9269	0.366588	0.05815	0.296937	0.041463	13.2225	0.349958
	Mean	1.83053	8.39525	6.58118	2.20568	5.74986	6.52952	8.7627	**1.82294**
${F}_{6}$	Best	1.00043	5.91488	3.60448	1.6921	2.8824	3.55587	**1.0E+00**	**1.0E+00**
	Std	1.30848	1.06214	1.72892	**0.31843**	1.69673	1.37074	3.38269	0.465247
	Mean	1268.01	1944.58	1084.19	1040.65	1086.12	716.955	1743.74	**653.691**
${F}_{7}$	Best	**302.563**	1489.48	349.742	549.339	481.84	470.187	1404.12	379.138
	Std	770.064	156.459	357.151	292.692	293.65	**124.894**	192.019	139.449
	Mean	5.05634	4.99653	4.29272	4.19395	4.34553	3.94024	4.627	**3.81781**
${F}_{8}$	Best	4.3575	4.76059	3.57905	**3.00511**	3.80226	3.60785	3.82306	3.44735
	Std	0.14473	**0.12244**	0.347452	0.416771	0.287821	0.167446	0.285393	0.185168
	Mean	**1.11521**	1.69237	1.37116	1.17887	1.3216	1.23611	1.11851	1.34592
${F}_{9}$	Best	1.03935	1.44267	1.14939	1.11338	1.11658	1.17482	1.07468	**1.03585**
	Std	0.047201	0.143125	0.124139	0.034882	0.127138	**0.03588**	0.017764	0.153126
	Mean	20.9988	21.3193	21.0069	21.0233	**19.7636**	21.1326	21.5252	20.9625
${F}_{10}$	Best	20.9802	21.1258	20.9958	20.9967	**1.14733**	20.6561	21.3316	17.7093
	Std	0.0739	0.116301	0.030266	**0.0037**	4.78486	0.113267	0.089198	0.714634

Significant values are in bold.

### Statistical analysis

To evaluate the proposed algorithm fairly and accurately, we perform statistical tests on the experimental results. To better determine whether the optimization results of GPOFWA were significantly different from those of other algorithms, a Wilcoxon nonparametric test was performed at a significance level of 0.05. A significance level $p$-value below 0.05 will be considered sufficient proof of the null hypothesis. The Wilcoxon tests for low dimensions (30 or less), 500 dimensions and CEC2019 are given in Tables 8, 9 and 10. In Tables 8, 9 and 10, values with $p$ greater than 0.05 are shown in bold, and NaN indicates that the result of the sum-of-values test is not a number. The last line shows the total counts in ($+/\approx /-$) format, where “$+$” indicates that the proposed GPOFWA outperforms the comparison algorithms at the 95% significance level (α = 0.05), ‘$-$’ indicates that the proposed GPOFWA algorithm exhibits poor performance in comparison, and “$\approx$” indicates that there is no significant statistical difference between the proposed GPOFWA algorithm and the comparison algorithm. From the last row, we can more intuitively compare the differences between different algorithms from a statistical point of view. From the last row of Table 8, it can be seen that GPOFWA outperforms other algorithms. We can conclude that from a statistical point of view, the performance of GPOFWA for low-dimensional function optimization is significantly different compared to other algorithms. Table 9 shows the Wilcoxon test results for the 500-dimensional function, and it is not difficult to see that the vast majority of $p$-values are less than 0.05 compared to other algorithms. It also shows that GPOFWA still has a statistically significant advantage on high-dimensional problems compared to other algorithms. Table 10 shows the Wilcoxon test results for the CEC2019 functions. It can be seen that except PPSO and HHOHGSO, GPOFWA still has obvious advantages compared with other algorithms.

**Table 8:** Statistical results of the Wilcoxon rank-sum test for low-dimension functions.

${F}_{n}$	HHO	GWO	SCA	SSA	WCA	WOA	LSA	PO
${F}_{1}$	1.21E−12	1.21E−12	1.21E−12	1.21E−12	1.21E−12	1.21E−12	1.21E−12	1.21E−12
${F}_{2}$	6.28E−06	3.02E−11	3.02E−11	3.02E−11	3.02E−11	3.69E−11	3.02E−11	1.47E−07
${F}_{3}$	1.21E−12	1.21E−12	1.21E−12	1.21E−12	1.21E−12	1.21E−12	1.21E−12	NaN
${F}_{4}$	1.21E−12	1.21E−12	1.21E−12	1.21E−12	1.21E−12	1.21E−12	1.21E−12	1.21E−12
${F}_{5}$	9.83E−08	9.83E−08	9.83E−08	9.83E−08	9.83E−08	9.83E−08	9.83E−08	9.83E−08
${F}_{6}$	NaN	1.17E−12	1.18E−12	1.19E−12	NaN	NaN	1.19E−12	NaN
${F}_{7}$	1.21E−12	1.21E−12	1.21E−12	1.21E−12	1.21E−12	1.21E−12	1.21E−12	NaN
${F}_{8}$	1.21E−12	1.21E−12	1.21E−12	1.21E−12	1.21E−12	1.21E−12	1.21E−12	1.21E−12
${F}_{9}$	NaN	1.21E−12	1.21E−12	1.21E−12	1.21E−12	1.21E−12	1.21E−12	NaN
${F}_{10}$	7.60E−07	3.02E−11	3.02E−11	3.02E−11	5.57E−10	3.02E−11	3.02E−11	4.56E−11
${F}_{11}$	1.21E−12	1.21E−12	1.21E−12	1.21E−12	1.21E−12	1.21E−12	1.21E−12	NaN
${F}_{12}$	1.21E−12	1.21E−12	1.21E−12	1.21E−12	1.21E−12	1.21E−12	1.21E−12	1.21E−12
${F}_{13}$	NaN	1.21E−12	1.21E−12	1.21E−12	1.69E−14	5.98E−05	1.21E−12	1.83E−08
${F}_{14}$	1.21E−12	1.21E−12	1.21E−12	1.21E−12	1.21E−12	1.21E−12	1.21E−12	NaN
${F}_{15}$	1.79E−11	8.48E−09	0.27719	3.02E−11	1.21E−12	5.57E−10	1.21E−12	3.16E−12
${F}_{16}$	1.87E−05	3.02E−11	3.02E−11	3.02E−11	3.02E−11	3.02E−11	3.02E−11	2.47E−08
${F}_{17}$	NaN	3.87E−10	1.21E−12	1.21E−12	1.21E−12	**0.33371**	1.21E−12	**0.33371**
${F}_{18}$	NaN	1.21E−12	1.21E−12	1.21E−12	1.03E−12	8.86E−07	1.11E−12	1.43E−06
${F}_{19}$	3.02E−11	3.02E−11	3.02E−11	3.02E−11	3.02E−11	3.02E−11	3.02E−11	3.02E−11
${F}_{20}$	1.21E−12	1.21E−12	1.21E−12	1.21E−12	1.21E−12	1.21E−12	1.21E−12	1.27E−05
${F}_{21}$	1.21E−12	1.21E−12	1.21E−12	1.21E−12	1.21E−12	1.21E−12	1.21E−12	1.21E−12
${F}_{22}$	NaN	4.98E−13	**0.33371**	1.21E−12	1.21E−12	3.14E−08	1.21E−12	NaN
${F}_{23}$	3.02E−11	3.02E−11	3.02E−11	3.02E−11	1.69E−09	3.02E−11	3.02E−11	1.21E−12
${F}_{24}$	1.21E−12	1.21E−12	1.21E−12	1.21E−12	6.24E−13	1.17E−12	1.20E−12	1.20E−12
${F}_{25}$	2.39E−08	3.02E−11	3.02E−11	3.02E−11	3.02E−11	3.02E−11	3.01E−11	1.21E−12
${F}_{26}$	NaN	0.021577	1.21E−12	1.21E−12	1.21E−12	**0.33371**	4.56E−12	NaN
${F}_{27}$	NaN	1.21E−12	1.21E−12	1.21E−12	1.21E−12	5.77E−11	1.21E−12	0.041774
${F}_{28}$	1.21E−10	3.02E−11	3.02E−11	3.02E−11	3.02E−11	3.02E−11	3.02E−11	2.84E−11
${F}_{29}$	1.41E−09	3.02E−11	3.02E−11	1.25E−07	3.02E−11	3.02E−11	**0.18577**	1.21E−12
${F}_{30}$	4.57E−09	3.02E−11	3.02E−11	3.02E−11	3.34E−11	3.02E−11	**0.12967**	1.21E−12
$+/\approx /-$	22/8/0	30/0/0	29/0/1	30/0/0	29/1/0	27/1/2	28/0/2	21/8/1

Significant values are in bold.

**Table 9:** Statistical results of the Wilcoxon rank-sum test for high-dimension functions.

${F}_{n}$	HHO	GWO	SCA	SSA	WCA	WOA	LSA	PO
${F}_{1}$	1.21E−12	1.21E−12	1.21E−12	1.21E−12	1.21E−12	1.21E−12	1.21E−12	NaN
${F}_{2}$	0.000201	3.02E−11	3.02E−11	3.02E−11	3.02E−11	1.46E−10	3.02E−11	7.77E−09
${F}_{3}$	1.21E−12	1.21E−12	1.21E−12	1.21E−12	1.21E−12	1.21E−12	1.21E−12	NaN
${F}_{4}$	1.21E−12	1.21E−12	1.21E−12	1.21E−12	1.21E−12	1.21E−12	1.21E−12	1.21E−12
${F}_{5}$	1.21E−12	1.21E−12	1.21E−12	1.21E−12	1.21E−12	1.21E−12	1.21E−12	1.21E−12
${F}_{6}$	1.96E−10	3.02E−11	3.02E−11	3.02E−11	3.02E−11	3.02E−11	3.02E−11	1.21E−12
${F}_{7}$	NaN	1.21E−12	**0.33371**	1.21E−12	1.21E−12	NaN	1.21E−12	NaN
${F}_{8}$	1.21E−12	1.21E−12	1.21E−12	1.21E−12	1.21E−12	1.21E−12	1.21E−12	1.21E−12
${F}_{9}$	NaN	1.21E−12	1.21E−12	1.21E−12	1.21E−12	1.21E−12	1.21E−12	NaN
${F}_{10}$	7.60E−07	3.02E−11	3.02E−11	3.02E−11	5.57E−10	3.02E−11	3.02E−11	4.56E−11
${F}_{16}$	0.002891	3.02E−11	3.02E−11	3.02E−11	3.02E−11	3.02E−11	3.02E−11	1.24E−09
${F}_{17}$	NaN	1.21E−12	1.21E−12	1.21E−12	1.21E−12	**0.33371**	1.21E−12	**0.16074**
${F}_{18}$	NaN	1.21E−12	1.21E−12	1.21E−12	1.21E−12	0.041774	1.21E−12	5.88E−08
${F}_{19}$	4.50E−11	3.02E−11	3.02E−11	3.02E−11	0.001953	3.02E−11	3.02E−11	3.02E−11
${F}_{20}$	1.21E−12	1.21E−12	1.21E−12	1.21E−12	1.21E−12	1.21E−12	1.21E−12	2.93E−05
${F}_{21}$	NaN	1.21E−12	1.21E−12	1.21E−12	1.21E−12	9.83E−08	1.21E−12	NaN
${F}_{22}$	4.50E−11	3.02E−11	3.02E−11	3.02E−11	3.02E−11	3.02E−11	3.02E−11	1.21E−12
${F}_{23}$	1.21E−12	1.21E−12	1.21E−12	1.21E−12	1.21E−12	NaN	1.21E−12	1.17E−12
${F}_{24}$	1.25E−05	3.02E−11	3.02E−11	3.02E−11	3.02E−11	3.02E−11	3.02E−11	4.56E−11
${F}_{25}$	NaN	1.21E−12	1.21E−12	1.21E−12	1.21E−12	1.21E−12	1.21E−12	NaN
$+/\approx /-$	14/6/0	20/0/0	19/0/1	20/0/0	20/0/0	17/2/1	20/0/0	13/6/1

Significant values are in bold.

**Table 10:** Statistical results of the Wilcoxon rank-sum test for CEC2019 functions.

${F}_{n}$	PO	CLPPSO	FWHHO	PPSO	HHOHGSO	DE	CMA-ES
${F}_{1}$	NaN	1.21E−12	2.93E−05	1.70E−08	NaN	1.21E−12	1.21E−12
${F}_{2}$	**0.59719**	1.72E−12	**0.0607**	9.74E−10	0.01996	1.72E−12	1.72E−12
${F}_{3}$	2.92E−09	5.49E−11	3.02E−11	0.003339	1.10E−08	3.02E−11	3.02E−11
${F}_{4}$	3.02E−11	3.02E−11	6.07E−11	**0.95873**	**0.10869**	4.08E−11	3.50E−09
${F}_{5}$	2.37E−10	3.02E−11	2.03E−07	**0.98231**	0.005555	3.02E−11	0.000446
${F}_{6}$	0.047928	4.50E−11	**0.04553**	1.60E−07	**0.46427**	4.42E−06	2.37E−10
${F}_{7}$	3.02E−11	2.20E−07	3.02E−11	**0.5106**	**0.97052**	4.80E−07	3.73E−07
${F}_{8}$	3.02E−11	1.86E−06	3.02E−11	1.39E−06	3.20E−09	0.013272	2.61E−10
${F}_{9}$	5.46E−09	7.09E−08	3.20E−09	**0.44642**	**0.5106**	6.36E−05	7.77E−09
${F}_{10}$	9.89E−08	4.12E−06	2.20E−07	2.78E−07	4.44E−07	**0.83026**	3.02E−11
$+/\approx /-$	8/1/1	10/0/0	8/0/2	6/0/4	5/1/4	9/0/1	10/0/0

Significant values are in bold.

### Convergence analysis

In original PO, the balance between the exploration and exploitation is attained through party switching, which uses a parameter λ to control the diversity, and the interaction between the constituency winners in the phase of parliamentary affairs ensures the convergence of PO^32^. CPOFWA adds many mechanisms on the basis of PO to enhance the performance of the algorithm. First, GPOFWA performs explosion spark and Gaussian explosion spark operations on party leaders and constituency winners based on greedy strategy, and the Gaussian explosion spark mechanism of the firework algorithm is used to explore areas with better fitness to ensure the effectiveness of RPPUS. The greedy strategy enhances exploitation capability of GPOFWA, and Gaussian spark for verification of RPPUS prevents excluding good solutions. In addition, Converged Mobility Center with bi-directional consideration enhances the exploitation ability and maintains the population diversity, avoiding local optima. We can also analyze the convergence of GPOFWA by observing the convergence curves of numerous test functions. It can be observed that GPOFWA has a faster convergence rate to produce accurate solutions in most cases compared to the comparison algorithms.

### Parameter sensitivity analysis

The GPOFWA mainly includes 4 parameters, which are the parameter $k$ that controls the number of sparks generated, the parameter $R$ that controls the radius of the spark explosion, the number of parties(constituencies) and initial party switching rate $\lambda$. Among them, the parameter $k$ and the parameter $k$ are unique to the GPOFWA. Therefore, we need to analyze the influence of parameters $k$ and $R$ on the performance of GPOFWA algorithm. Experiments were conducted under four sets of parameters in Table 11. The number of parties (constituencies) is set to 8 and the initial party conversion rate λ is set to 1. We selected several unimodal functions (F2 and F6), multimodal functions (F16 and F23), and fixed dimension functions (F28 and F29) as representatives to test the performance of the algorithm under different parameters. The statistical results of GPOFWA are shown in Table 11, and the best results are shown in bold. According to Table 9, when $k=50$ and $R=50$, the number of optimal values obtained is 5, which is greater than the number of other cases. Hence, $k=50$ and $R=50$, is the best choice of parameters.

**Table 11:** Statistical results with different $k$ and $R$ values.

${F}_{n}$	$k=50 \& R=40$	$k=50 \& R=50$	$k=40 \& R=40$	$k=40 \& R=50$
${F}_{2}$	**5.20E−06**	5.54E−06	8.87E−05	1.58E−05
${F}_{6}$	**5.35E−08**	1.15E−07	6.59E−08	1.27E−07
${F}_{16}$	6.95E−05	1.05E−04	2.34E−04	**1.76E−05**
${F}_{23}$	**5.28E−216**	4.52E−215	4.15E−215	4.46E−215
${F}_{28}$	**0.0E+00**	2.35E−265	3.58E−265	**0.0E+00**
${F}_{29}$	**3.0E+00**	7.84025	3.00001	3.00003
Number of winners	5	0	0	2

Significant values are in bold.

## Engineering optimization problems

In this section, we apply GPOFWA to three well- known constrained engineering problems: welded beam design problem, spring design problem and three bar truss problem to demonstrate its performance in solving practical problems. For the fairness and rationality of the experiment, each experiment is independently run 30 times and the number of iterations is 500. These engineering problems are abstracted from various scenes in the real world, which are composed of an objective function and multiple constraints. Therefore, we need a suitable method to deal with these constraint conditions in these engineering problems. In this section, we employ the penalty function method. In this approach, solutions which violate any of the constraints are penalized by a large fitness value (in case of minimization). The penalty function is defined as follows:

20 $$\begin{array}{*{20}c} {F(x) = f(x) + \lambda *\mathop \sum \limits_{i = 1}^{p} \left\{ {\max \left[ {0,{ }g_{i} (x)} \right]} \right\} + \lambda *\mathop \sum \limits_{i = 1}^{q} \left\{ {\max \left[ {0,{ }\left| {h_{j} (x)} \right|} \right]} \right\}} \\ \end{array}$$

where $\lambda$ is penalty factor, and it is initialized to $10^{10}$ in this section.

### Welded beam design problem

**Figure 11:**
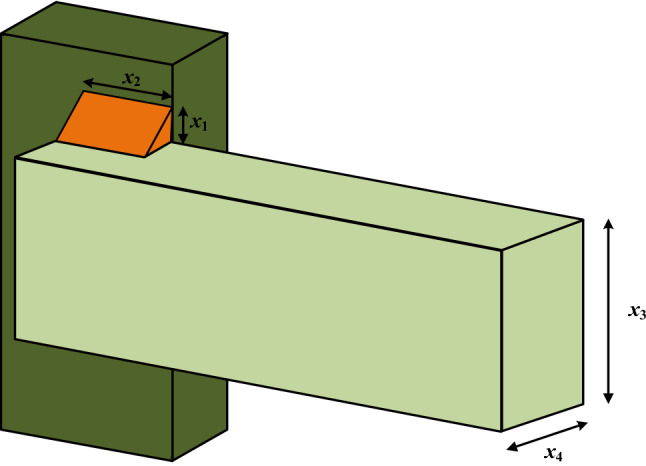
Welded beam design problem.

The goal of the welded beam design problem is to determine the best cost of welding beams with strong members. As shown in Fig. 11, there are four parameters that can be optimized for welded beam: height (*h*), length (*l*), weld thickness (*t*) and thickness (*b*). Its constraints consist of shear ($\tau$), beam blending stress ($\sigma$), bar bucking load ($P_{c}$) and beam end deflection ($\delta$) and side constraints. The mathematical expression of WBD problem is given by:

$$\begin{array}{*{20}l} {{\text{Consider}}} \hfill & {\vec{l} = \left[ {l_{1} l_{2} l_{3} l_{4} } \right] = \left[ {hltb} \right] = \left[ {x_{1} x_{2} x_{3} x_{4} } \right],} \hfill \\ {{\text{minimize}}} \hfill & {f\left( {\vec{l}} \right) = l_{1}^{2} l_{2} *1.10471 + 0.04811*l_{3} l_{4} *\left( {14.0 + l_{2} } \right),} \hfill \\ {{\text{Subject}}\;{\text{to}}} \hfill & {s_{1} \left( {\vec{l}} \right) = \tau \left( {\vec{l}} \right) - \tau_{{{\text{max}}}} \le 0,} \hfill \\ {} \hfill & {s_{2} \left( {\vec{l}} \right) = \sigma \left( {\vec{l}} \right) - \sigma_{{{\text{max}}}} \le 0,} \hfill \\ {} \hfill & {s_{3} \left( {\vec{l}} \right) = \delta \left( {\vec{l}} \right) - \delta_{{{\text{max}}}} \le 0,} \hfill \\ {} \hfill & {s_{4} \left( {\vec{l}} \right) = l_{1} - l_{4} \le 0,} \hfill \\ {} \hfill & {s_{5} \left( {\vec{l}} \right) = {{\rm P}} - P_{c} \left( {\vec{l}} \right) \le 0,} \hfill \\ {} \hfill & {s_{6} \left( {\vec{l}} \right) = 0.125 - l_{1} \le 0,} \hfill \\ {} \hfill & {s_{7} \left( {\vec{l}} \right) = 1.10471*l_{1}^{2} + 0,0481*l_{3} l_{4} \left( {14.0 + l_{2} } \right) - 5.0 \le 0,} \hfill \\ \end{array}$$

Decision variable interval values:

$$\begin{aligned} & 0.1 \le l_{1} \le 2, \\ & 0.1 \le l_{2} \le 10, \\ & 0.1 \le l_{3} \le 10, \\ & 0.1 \le l_{4} \le 2, \end{aligned}$$

where

$$\begin{aligned} \tau \left( {\vec{l}} \right) & = \sqrt {\tau^{{\prime}2} + 2\tau^{{\prime}} \tau^{{\prime\prime}} \left( {\frac{{l_{2} }}{R}} \right) + \left( {\tau^{{\prime\prime}} } \right)^{2} } , \\ \tau^{{\prime}} & = \frac{{{\rm P}}}{{\sqrt 2 l_{1} l_{2} }}, \tau^{{\prime\prime}} = \frac{{{{{\rm M}{\rm H}}}}}{{{\rm N}}},\quad {{\rm M}} = \left( {{{\rm K}} + \frac{{l_{2} }}{2}} \right), \\ {{\rm H}} & = \sqrt {\frac{{l_{2}^{2} }}{4} + \left( {l_{1} + \frac{{l_{3} }}{2}} \right)^{2} } , \\ {{\rm N}} & = 2\left\{ {\sqrt 2 l_{1} l_{2} \left[ {\left( {\frac{{l_{2}^{2} }}{4} + \left( {l_{1} + \frac{{l_{3} }}{2}} \right)} \right)^{2} } \right]} \right\}, \\ P_{c} \left( {\vec{l}} \right) & = \frac{{\frac{{4.013{{\rm E}}\sqrt {l_{3}^{2} l_{4}^{6} } }}{36}}}{{{{\rm K}}^{2} }}\left( {1 - \frac{{\frac{{l_{3} }}{{2{{\rm K}}}}\sqrt {{\rm E}} }}{4G}} \right), \\ \end{aligned}$$

where $\sigma_{{{\text{max}}}} = 30000$ psi, *P* = 6000 lb, L = 14 in, $\delta_{{{\text{max}}}} = 0.25$ in, ${{\rm E}} = 3 \times 10^{6}$ psi, $\tau_{{{\text{max}}}} = 13600$ psi and $G = 12 \times 10^{6}$ psi.

We compare the statistical results of 30 independent executions of GPOFWA with some other excellent algorithms, and show the values of the design variables obtained, the mean, best value and variance of the optimal solution in Table 12. The results show that the performance of GPOFWA is better than other algorithms.

**Table 12:** Comparison of GPOFWA with other algorithms for the welded beam design problem.

Algorithm	*h*	*l*	*t*	*b*	Mean	Best	SD
GPOFWA	0.2057	3.2530	9.0366	0.2057	**1.69524**	**1.69524**	**1.78E−09**
PO	0.2058	3.2529	9.3040	0.2059	1.70073	1.69625	4.85E−03
HHO	0.1984	3.3952	9.0113	0.2097	1.87785	1.72912	0.12328
GWO	0.2051	3.2647	9.03	0.2057	1.69856	1.69622	2.14E−03
SCA	0.1991	3.4173	9.3313	0.2066	1.84615	1.76477	0.04143
SSA	0.2016	3.3195	9.0697	0.2056	1.81378	1.70251	0.11945
WCA	0.2057	3.2530	9.0366	0.2057	**1.69524**	**1.69524**	5.46E−08
WOA	0.2023	3.1573	9.6389	0.2035	2.20313	1.76203	0.46774
LSA	0.2085	3.1913	9.3928	0.2085	1.90137	**1.69524**	0.19221

Significant values are in bold.

### Spring design problem

**Figure 12:**
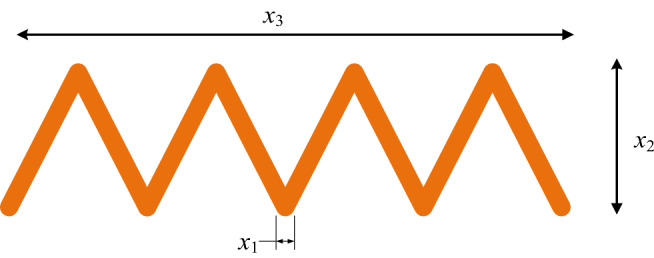
Speed reducer beam design problem.

This constrained engineering problem is to design a tension/compression spring with minimum weight, the structure of which is shown in Fig. 12. There are three variables that can be optimized, including the diameter of the wire (*d*), coil (*D*) and the number of the active coil (*N*). The Spring design problem is mathematically formulated as follows:

$$\begin{array}{*{20}l} {{\text{Consider}}} \hfill & {\vec{l} = \left[ {l_{1} l_{2} l_{3} } \right] = \left[ {dDN} \right] = \left[ {x_{1} x_{2} x_{3} } \right],} \hfill \\ {{\text{Minimize}}} \hfill & {f\left( {\vec{l}} \right) = \left( {l_{3} + 2} \right)*l_{2} l_{1}^{2} ,} \hfill \\ {{\text{Subject}}\;{\text{to}}} \hfill & {s_{1} \left( {\vec{l}} \right) = 1 - \frac{{l_{2}^{3} l_{3} }}{{717851^{4} }} \le 0,} \hfill \\ {} \hfill & {s_{2} \left( {\vec{l}} \right) = \frac{{4l_{2}^{2} - l_{1} l_{2} }}{{12566\left( {l_{3} l_{1}^{3} - l_{1}^{4} } \right)}} + \frac{1}{{5108l_{1}^{2} }} \le 0,} \hfill \\ {} \hfill & {s_{3} \left( {\vec{l}} \right) = 1 - \frac{{140.45l_{1} }}{{l_{2}^{2} l_{3} }} \le 0,} \hfill \\ {} \hfill & {s_{4} \left( {\vec{l}} \right) = \frac{{l_{2} + l_{1} }}{1.5} - 1 \le 0,} \hfill \\ \end{array}$$

Decision variable interval values:

$$\begin{aligned} & 0.05 \le l_{1} \le 2.00, \\ & 0.25 \le l_{2} \le 1.30, \\ & 2.00 \le l_{3} \le 15.0, \\ \end{aligned}$$

We also compare the statistical results of 30 independent executions of GPOFWA with some other excellent algorithms, and show the values of the design variables obtained, the mean, best value and variance of the optimal solution in Table 13. The results show that GPOFWA can get better results than other algorithms. GPOFWA has performed well in these two engineering application problems, which shows that the algorithm better balance the relationship between exploration and exploitation.

**Table 13:** Comparison of GPOFWA with other algorithms for the spring design problem.

Algorithm	*d*	*D*	*N*	Mean	Best	SD
GPOFWA	0.1391	1.3000	11.8924	**3.66189**	**3.66189**	**1.63E−15**
PO	0.1391	1.3000	11.8924	3.67894	**3.66189**	2.53E−07
HHO	0.1391	1.3000	11.8924	3.69192	**3.66189**	0.02796
GWO	0.1391	1.3000	11.8924	3.66191	**3.66189**	1.44E−05
SCA	0.1392	1.3000	11.9196	3.68802	3.66244	0.02071
SSA	0.1392	1.3000	11.9035	3.67936	3.66285	0.01743
WCA	0.1391	1.3000	11.8924	3.66825	**3.66189**	2.11E−15
WOA	0.1392	1.3000	11.9063	3.69615	3.68346	0.02911
LSA	0.1391	1.3000	11.8924	**3.66189**	**3.66189**	2.20E−15

Significant values are in bold.

### Three bar truss design problem

**Figure 13:**
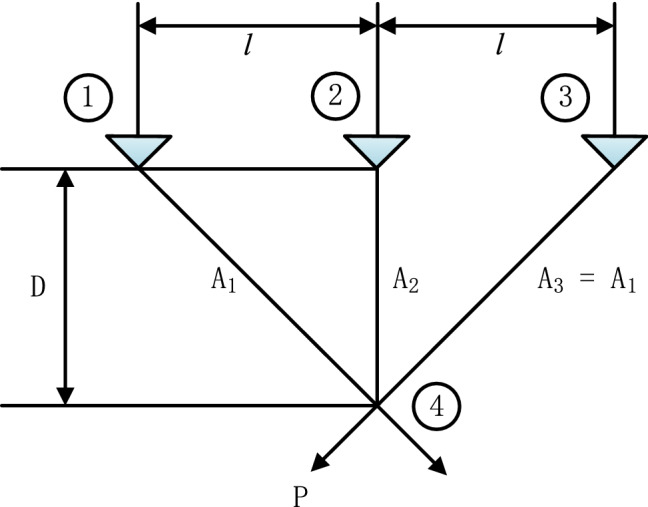
Three bar truss design problem.

The threE−bar truss design problem is a classic design problem in the field of engineering structure. The optimization goal of this design problem is to design a truss as light as possible, which must meet the three constraints of stress, deflection and buckling. This problem aims to minimize the volume of the truss structure subject to 3 stress constraints. The structural model and parameters of the threE−bar truss design problem are shown in the Fig. 13 and the mathematical formulation of this problem is given below:

$$\begin{array}{*{20}l} {{\text{Consider}}} \hfill & {\vec{x} = \left[ {x_{1} x_{2} } \right] = \left[ {A_{1} A_{2} } \right],} \hfill \\ {{\text{Minimize}}} \hfill & {f\left( {\vec{x}} \right) = \left( {2\sqrt {2x_{1} + x_{2} } } \right)l,} \hfill \\ {{\text{Subject}}\;{\text{to}}} \hfill & {\frac{{\sqrt {2x_{1} } + x_{2} }}{{\sqrt {2x_{1}^{2} } + 2x_{1} x_{2} }}p - \sigma \le 0,} \hfill \\ {} \hfill & {\frac{{x_{2} }}{{\sqrt {2x_{1}^{2} } + 2x_{1} x_{2} }}p - \sigma \le 0,} \hfill \\ {} \hfill & {\frac{1}{{\sqrt {2x_{1}^{2} } + x_{1} }}p - \sigma \le 0,} \hfill \\ \end{array}$$

Decision variable interval values:

$$\begin{aligned} & 0 \le x_{1} \le 1, \\ & 0 \le x_{2} \le 1, \\ & l = 100, \\ & p = 20, \\ & \sigma = 2.0, \\ \end{aligned}$$

We compare the statistical results of 30 independent executions of GPOFWA with other excellent algorithms, and show the values of the design variables obtained, the mean, best value and variance of the optimal solution in Table 14. The results show that the optimal values of GPOFWA and PO, SSA and WCA are consistent, but the average and variance of GPOFWA are the smallest among all algorithms, which indicates that the proposed GPOFWA is feasible and effective for solving the design problem of threE−bar truss.

**Table 14:** Comparison of GPOFWA with other algorithms for threE−bar truss design problem.

Algorithm	${A}_{1}$	${A}_{2}$	Mean	Best	SD
GPOFWA	0.7867	0.2880	**186.3859**	**186.3859**	**3.57E−14**
PO	0.7868	0.2884	186.3860	**186.3859**	3.42E−05
HHO	0.7879	0.2939	186.3976	186.3860	0.0235
GWO	0.7869	0.2879	186.3860	186.3860	3.89E−05
SCA	0.7821	0.2970	186.8456	186.4058	2.4609
SSA	0.7868	0.2880	**186.3859**	**186.3859**	2.69E−08
WCA	0.7869	0.2880	186.3860	**186.3859**	2.31E−10
WOA	0.7901	0.2896	186.7263	186.3940	0.2165
LSA	0.7868	0.2898	186.4503	186.3922	5.08E−09

Significant values are in bold.

## Conclusions

As an emerging swarm intelligence algorithm, PO has good exploration capability, exploration capability, and convergence speed, but the subgroup optimal solution used by the original PO is limited, and PO’s recent past-based position updating strategy (RPPUS) has loopholes. The explosion search mechanism of the firework algorithm has certain potential and unique advantages. In this paper, the explosion search mechanism of the firework algorithm is used to expand and optimize the subgroup optimal solution in the political optimization algorithm. At the same time, the Gaussian explosion spark of the firework algorithm is used to make up for some of the shortcomings of RPPUS. In addition, a new local leader called Converged Mobile Center (CMC) based on two-way consideration was designed to guide the movement of search agents.

Based on these, a hybrid algorithm called GPOFWA is obtained. In order to verify the good performance of GPOFWA, we conducted a two-part experiment. In the first part, we selected a set of well-researched different benchmark functions and compared them with new swarm intelligence optimization algorithms including the original HHO, GWO, SCA, SSA, WCA, WOA, LSA, PO. Compared with PO, this algorithm has significantly improved accuracy, convergence curve, stability, and robustness when solving functions that are unimodal or multimodal. Compared with other methods, GPOFWA also shows significant advantages. In the second part, we apply GPOFWA to three constrained engineering problems, because of the improvement of the explosion search mechanism, GPOFWA can achieve the best results in all engineering design problems. The results show that GPOFWA has excellent performance for engineering design problems, and it is believed that GPOFWA can expect the same performance for other more complex engineering problems.

In addition to the qualities mentioned above, PO has some limitations that need to be highlighted. The limitations of GPOFWA are as follows: Due to the addition of the explosive search mechanism, the algorithm time overhead has increased, although CMC has reduced this newly added time overhead as much as possible. Second, the algorithm has a total of 4 parameters, which is relatively complex and needs to be improved in the future. In future work, the GPOFWA algorithm can also consider a binary version to solve discrete practical problems, such as antenna design, feature selection, etc. At the same time, we can also combine CMC with other swarm optimization algorithms to further test its performance.

## Data Availability

All data generated or analyzed during this study are included in this article.

## Code availability

The code used to evaluate the proposed algorithm GPOFWA is available with the paper. Full codes are available from the authors upon reasonable request.
